# Epstein–Barr Virus, Lower Vitamin D, Low Sun Exposure, and HLA‐DRB1*1501 Risk Variant Share Common Epigenetic Pathways Leading to Multiple Sclerosis Onset

**DOI:** 10.1002/ana.78043

**Published:** 2025-10-10

**Authors:** Steve Simpson‐Yap, Ellen Morwitch, Samuel A. Tanner, Sarah M. Thomson, Alex Eisner, Rod A. Lea, Trevor J. Kilpatrick, Jeannette Lechner‐Scott, Rodney J. Scott, Alexandre Xavier, Vicki E. Maltby, Robyn M. Lucas, Bruce V. Taylor, Brett A. Lidbury, Simon A. Broadley, Ingrid van der Mei, Mehari Woldemariam Merid, Boris Novakovic, Richard Saffery, Anna Karin Hedström, Pernilla Stridh, Tomas Olsson, Maja Jagodic, Lars Alfredsson, Anne‐Louise Ponsonby, for the Ausimmune Investigator Group, Caron Chapman, Trevor Kilpatrick, Robyn Lucas, Tony McMichael, Anne‐Louise Ponsonby, Bruce Taylor, Ingrid van der Mei, David Williams, Alan Coulthard, Keith Dear, Terry Dwyer, Patricia Valery

**Affiliations:** ^1^ The Florey Institute of Neuroscience and Mental Health, The University of Melbourne Parkville VIC Australia; ^2^ Neuroepidemiology Unit, Melbourne School of Population & Global Health, The University of Melbourne Carlton VIC Australia; ^3^ MS Research Flagship, Menzies Institute for Medical Research, University of Tasmania Hobart TAS Australia; ^4^ Murdoch Children's Research Institute, The University of Melbourne Parkville VIC Australia; ^5^ Melbourne Bioinformatics, The University of Melbourne Parkville VIC Australia; ^6^ Centre for Genomics and Personalised Health, School of Biomedical Science, Queensland University of Technology Kelvin Grove QLD Australia; ^7^ School of Medicine and Public Health, The University of Newcastle Callaghan NSW Australia; ^8^ Hunter Medical Research Institute, The University of Newcastle Callaghan NSW Australia; ^9^ Department of Neurology, John Hunter Hospital New Lambton Heights NSW Australia; ^10^ School of Biomedical Sciences and Pharmacy, The University of Newcastle Callaghan NSW Australia; ^11^ National Centre for Epidemiology and Population Health, The Australian National University Acton ACT Australia; ^12^ School of Medicine and Dentistry, Gold Coast Campus, Griffith University Southport QLD Australia; ^13^ Department of Epidemiology and Biostatistics Institute of Public Health, College of Medicine and Health Sciences, University of Gondar Gondar Ethiopia; ^14^ Department of Paediatrics The Royal Children's Hospital Parkville VIC Australia; ^15^ Department of Clinical Neuroscience Karolinska Institutet Stockholm Sweden; ^16^ Center for Molecular Medicine, Karolinska University Hospital Stockholm Sweden; ^17^ Institute of Environmental Medicine, Karolinska Institutet Stockholm Sweden

## Abstract

**Objectives:**

Multiple sclerosis (MS) onset risk factors include Epstein–Barr virus (EBV) indices (including host response), lower serum 25‐vitamin D (25(OH)D) levels, low sun exposure, and HLA‐DRB1*1501. The underlying molecular mechanisms are unclear. Here, we examined mediation through differential DNA methylation (DNAm) to better understand possible epigenetic programming.

**Methods:**

Two case‐control studies (Ausimmune Study, Australia = 206 cases + 348 controls; and Epidemiologic Investigations of MS [EIMS], Sweden = 140 cases + 139 controls). DNAm was measured using Illumina arrays. Dimension‐reduction methods generated MS‐associated DNAm modules. Pathway enrichment analyses were used to describe DNAm modules’ system‐level biological characteristics. Individual and joint associations with MS risk were assessed using logistic regression. DNAm module mediation of risk factor‐outcome associations were assessed using mediation analysis. A range of temporality analyses were used.

**Results:**

EBV indices (infectious mononucleosis history and anti‐EBNA IgG titer), lower 25(OH)D, low sun exposure, and HLA‐DRB1*1501 risk variant were individually and jointly associated with MS risk. In each study, 2 DNAm modules were found which mediated multiple exposure‐MS associations. Proportions mediated ranged from 21 to 47% in Ausimmune and 25 to 53% in EIMS. Results were robust to sensitivity analyses. Top‐ranked genomewide association study (GWAS) MS risk‐associated genes were over‐represented in both Ausimmune DNAm modules, A1 3.5‐fold (*p* = 0.004) and A2 3‐fold (*p* = 0.015). Reactome pathways enriched for DNAm had cross‐study overlap – 45% of pathways enriched in Ausimmune DNAm modules were also enriched in EIMS (4.82‐fold, *p* < 0.001).

**Interpretation:**

EBV, lower vitamin D, low sun exposure, and HLA‐DRB1*1501 risk variant act in concert and through common epigenetic pathways to impact MS onset risk. ANN NEUROL 2026;99:341–355

## Graphical abstract

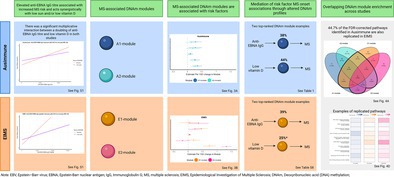

Multiple environmental and genetic risk factors for multiple sclerosis (MS) onset risk have been identified.^1^ Infection and associated host response to Epstein–Barr virus (EBV), the causative agent for infectious mononucleosis (IM), are the strongest and most replicated risk factors for MS, with self‐reported IM history and host anti‐EBV immune response (higher anti‐EBNA IgG titer) each independently associated with elevated risk.^2^ Other MS risk factors include low vitamin D status (as measured by serum 25‐hydroxyvitamin D [25(OH)D] levels) and low sun exposure, and the HLA‐DRB1*1501 locus encoding major histocompatibility complex class II genes.^3^

Not all EBV seropositive people develop MS, indicating co‐factors may play an important role.^4^ Component causes of a common pathway often exhibit interaction, where the disease effects of one factor varies depending on another.^5^ Such interactions have been demonstrated between EBV and other MS risk factors,^6^ and, in addition, gene–environment interactions have been demonstrated in MS risk, including among EBV, HLA‐DRB1*1501 risk genotype (AA/AG vs GG; hereafter referred to as HLA‐DRB1*1501), and other factors.^7^, ^8^ These results support the multifactorial nature of MS risk and the interdependency between risk factors. However, the underlying mechanisms remain unclear.

Epigenetics, such as DNA methylation (DNAm), refers to heritable but modifiable mechanisms of genetic regulation that represent an interface for environmental and genetic factors to influence the epigenome. DNAm refers to the addition of a methyl group to a DNA nucleotide (usually the cytosine of a cytosine‐guanine pairing [CpG]).^9^, ^10^ People with various diseases, including MS, have epigenetic DNAm profiles that differ compared with healthy controls. MS cases have altered DNAm in a range of genes in immune cell populations^11^, ^12^, ^13^ and in the central nervous system (CNS).^14^ Peripheral blood DNAm profiles have been correlated with clinical phenotype in MS^15^ and change with treatment.^16^, ^17^ We demonstrated differential DNAm in whole‐blood between MS cases recruited at disease onset (first clinical diagnosis [FCD]) and matched controls in the Ausimmune Study,^18^ an Australian multicenter case‐control study, with external validation in the Swedish Epidemiologic Investigations in MS (EIMS) case–control study.^6^

These findings indicate that MS risk factors may induce epigenetic programming as part of MS pathogenesis. Mediation occurs when a risk factor exerts an effect on an outcome through a mediating factor.^19^ DNAm is known to be responsive to environmental and genetic factors. For example, the HLA‐DRB1*1501 has been shown to impact on MS, in part, through differential DNAm.^20^, ^21^, ^22^ Environmental factors are also candidates to induce epigenetic alteration and increase MS but have not yet been investigated in depth. One candidate is EBV and the associated host response as both are associated with DNAm alterations.^23^ In another autoimmune disease, rheumatoid arthritis, a majority of the gene–environment interaction between smoking and a single nucleotide polymorphism (SNP) in the HLA locus (rs6933349) was found to be mediated by differential DNAm at that same SNP location (cg21325723).^24^ Further, it is possible that multiple genetic and environmental factors act through common epigenetic pathways. Therefore, it is important to examine how epigenetic alterations may mediate not only single risk factors in MS but also joint effects.

Dimension‐reduction approaches, which can generate DNAm modules, including sets of functionally related CpGs, operating via shared pathways,^25^ represents a potentially very useful approach.^26^ Badam et al derived a multi‐omic module that included 217 genes differentially methylated in MS, these genes were enriched for immune signaling pathways and also overlapped with 5 established MS risk factors.^25^ Whereas this study did not undertake a formal mediation analysis by DNAm of these risk factor‐MS associations, it represents a strong justification for this system biology approach.

Here, we analyzed data from the Ausimmune^18^ and EIMS^21^ case–control studies to (i) describe individual and joint effects of exposure associations with MS risk; (ii) construct MS‐associated DNAm modules and evaluate their associations with key MS risk factors; (iii) perform mediation analysis using DNAm modules as mediators of exposure‐MS associations; and (iv) evaluate the biological characteristics of mediating DNAm modules using pathway‐enrichment approaches.

## Materials and Methods

### Study Characteristics

#### The Ausimmune Study (2003–2007)

This was a multicenter incident matched case–control study across 4 regions of eastern Australia: Brisbane City, Queensland, Newcastle City and surrounds, New South Wales, Geelong and Western Districts of Victoria, Victoria, and the state of Tasmania.^18^ Cases (91% response rate) were recruited soon after FCD (median time interval = 148, interquartile range = 78 to 200 days).^27^ Controls (60% response rate of those contacted) were randomly selected from the compulsory Australian Electoral Roll, matched to cases by age (± 2 years), sex, and study region.^27^

Here, we restricted to FCD cases (n = 206) who did not have a non‐MS diagnosis by the 10‐year follow‐up. Of these, 93% (n = 192) had a recorded clinical or radiological evidence substantiating a clinically definite MS (CDMS) diagnosis (in keeping with 2010 McDonald criteria^28^) and 7% (n = 14) were lost to follow‐up. We utilized matched controls (n = 348) with DNAm data available. FCD cases were classified by neurologist review at study entry as: “first demyelinating event (FDE)” with no prior undiagnosed episodes of suspected demyelination (n = 107), FCD with suspected prior undiagnosed episodes (n = 84), and progressive‐onset cases (n = 15). Ausimmune was approved by 9 regional human research ethics committees and all participants provided written informed consent.

At their study clinic, participants completed comprehensive surveys querying a number of environmental and other factors over the life course,^18^ including self‐reported history of IM (as well as the age periods at which this occurred), and a life calendar‐based assessment of summer and winter sun exposures, from which seasonal sun exposure frequencies in the 3 years prior to the study were derived (dichotomized at ≥ 2 hours/day). Sociodemographic factors, diet, smoking, and supplement use were also recorded. Medication use, including disease‐modifying therapies (DMTs), was also recorded.^18^, ^29^ At this study clinic, biospecimens were collected from each participant using phlebotomy. From these, serum 25(OH)D concentrations were measured using liquid chromatography tandem mass‐spectrometry^27^ and seasonally adjusted,^30^ and serum anti‐EBNA IgG titers were measured using immunofluorescence assay (dichotomized at ≥ 640).^29^ DNA was extracted from whole‐blood using QIAamp DNA Blood Mini kit (Qiagen, The Netherlands). Genotyping was conducted by the Global Screening Array version 2.1 (Illumina, USA). DNAm was measured using the Illumina Infinium Human Methylation EPIC BeadChip Kit (Illumina, USA) and processed as described previously, resulting in 742,961 CpGs included for analysis.^22^

#### Epidemiological Investigation of Multiple Sclerosis (2005‐2011)

This was a Swedish MS case–control study, matched on age, sex, and ethnicity.^6^ Controls were selected from a national Swedish population register.^6^ EIMS response rates for cases and controls were 93% and 70%, respectively.^6^ The smaller participant sample used for EIMS DNAm analyses (n = 140 cases and 139 controls) were matched on age, sex, ethnicity (all Swedish), and smoking status (never/ex‐smoker/current). Cases were diagnosed according to the 2010 McDonald criteria.^28^ EIMS was approved by the Regional Ethical Review Board at Karolinska Institutet, and all participants provided written informed consent.

At the EIMS study clinic, participants were queried for a range of environmental factors, including IM history and sun exposure behaviors using standardized questionnaires.^6^ Sun exposure in the 5 years prior to the date of interview was based on a composite score from 3 questions: (1) frequency of sunbathing, (2) frequency of recent travel to a sunnier country than Sweden, and (3) frequency of use of sunbeds, estimating a total score ranging between 3 and 12 and dichotomized at > 6.^6^ Medication use, including DMTs, was recorded. At this same clinic, biospecimens were collected from each participant using phlebotomy, from which serum anti‐EBNA EBIgG (EBV‐EBNA‐peptide segment, aa385‐420) was measured using multiplex serological assay^31^, ^32^ (dichotomized at median = 6716) and serum 25(OH)D estimated using chemiluminescent immunoassay (Diasorin).^33^

HLA genotypes were measured using the Illumina exome chip (Illumina, USA), supplemented by HLA*IMP:02 imputation.^34^ DNAm was measured using the Illumina 450 K array (Illumina, USA), and 478,687 CpGs were included for analysis.^22^ This DNAm dataset is available on the Gene Expression Omnibus (GEO) database (GSE43976 and GSE106648).

### Statistical Methods

**FIGURE 1:**
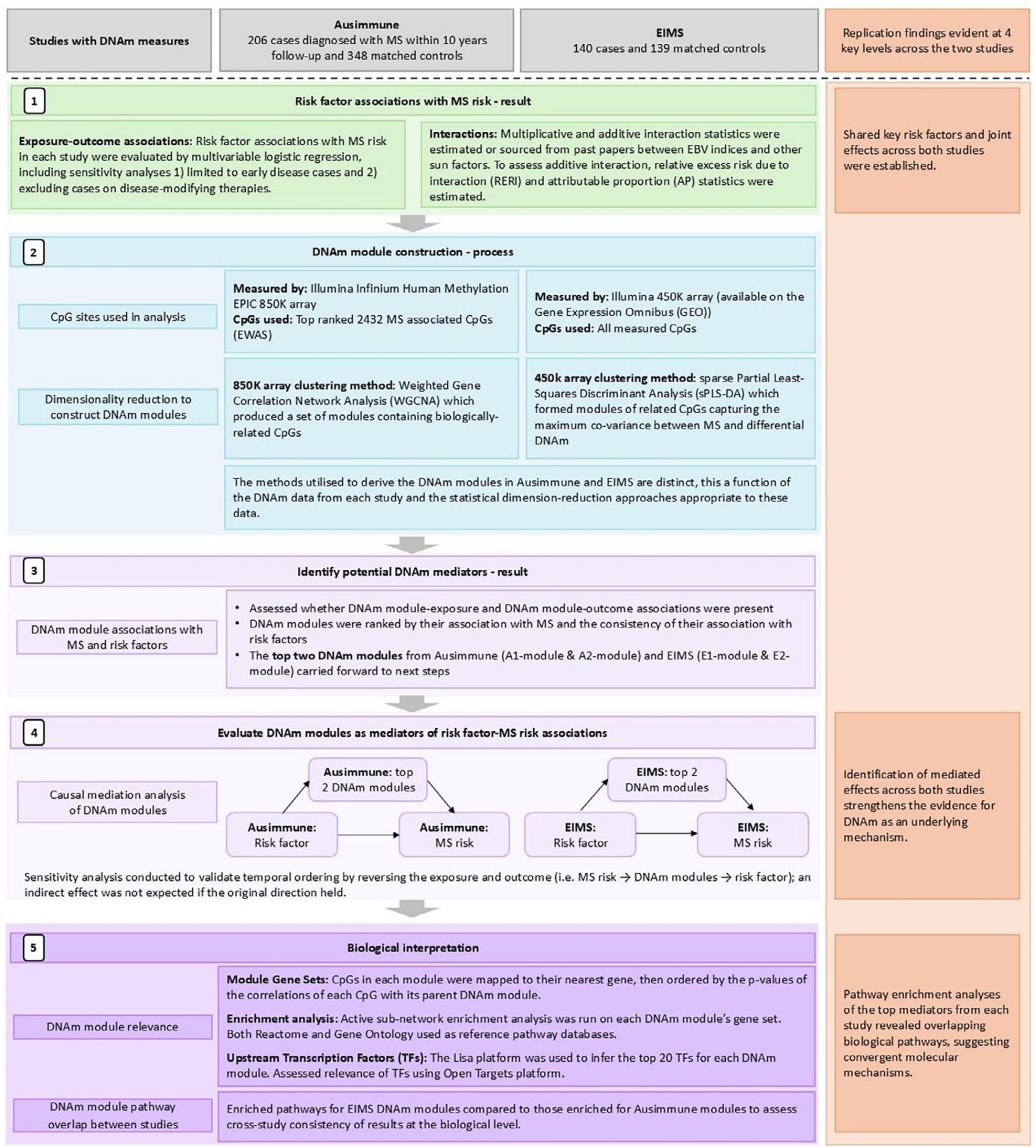
Visual summary and outline of analytical steps used in the project analyses. Analysis of the 2 study datasets included here (Ausimmune and EIMS) were limited in each to those participants with DNAm data, and in Ausimmune also to those cases who converted to MS by 10‐year follow‐up and their associated controls. (1) Analyzed exposure associations with MS risk in each study dataset, including individual and joint (additive and multiplicative interaction effects. (2) Derived DNAm modules utilizing DNAm data from each study and dimension‐reduction applied to each. (3) Evaluated DNAm associations with MS risk and with exposures, to determine those suitable for use as mediators. (4) Evaluated DNAm modules as mediators of exposure‐MS risk associations. (5) Determined the characteristics of DNAm modules using sub‐network enrichment and transcription factor analyses. DNAm = DNA methylation; EIMS = Epidemiologic Investigations of Multiple Sclerosis; MS = multiple sclerosis. [Color figure can be viewed at www.annalsofneurology.org]

The series of analytical steps are summarized in Figure 1. Analyses were conducted in R software (version 4.3.2). Further details on the 5 main components are outlined below. All analyses were complete case.

Exposure‐outcome associations were assessed by multivariable logistic regression, adjusted for matching factors (Ausimmune = age, sex, and region and EIMS = age, sex, ethnicity, and smoking status). Because smoking was a matching criterion in the EIMS DNAm substudy, it could not be examined as a risk factor here.

#### Interactions

We examined additive and multiplicative interactions between each of IM, anti‐EBNA IgG titer, vitamin D, and sun exposure and HLA‐DRB1*1501 in their associations with MS case status in Ausimmune.^35^, ^36^ Categorical terms from dichotomized exposures were used to assess additive interaction between EBV indices (IM history and anti‐EBNA IgG titer) and low vitamin D or low sun, as well as between EBV indices and HLA‐DRB1*1501 genotype. Relative excess risk due to interaction (RERI) and attributable proportion due to interaction (AP) statistics were estimated. Assessment of statistical trends (p_trend_) was done by evaluating ordered polychotomous terms and reporting the *p* value from these terms.

#### Network‐Level Investigations

These analyses had a focus on system‐level epigenomics.^25^, ^37^ We constructed DNAm modules, which are MS‐associated networks of CpGs formed by well‐known clustering algorithms. We then assessed the biological characteristics of each module by pathway enrichment analysis and the inference of upstream transcription factors. Such functionally connected modules were then examined in relation to both MS and risk factors and tested using counterfactual mediation analysis.^38^ We then assessed system‐level replication of our results based on the consistencies of the enriched pathways across the studies.

#### DNAm Module Construction in Ausimmune

To ensure MS‐specific DNAm results, we first ranked CpGs by association with MS onset risk in an epigenome‐wide association study (EWAS) using a cross‐package Bioconductor workflow.^39^ An adjustment was made for cell type proportion using the EpiDish R package,^40^ ancestry (first 2 principal components),^22^ and study matching covariates. The top‐ranked CpGs (n = 2,432, Benjamini‐Hochberg adjusted *p* value,^41^ p_adjusted_ < 5 × 10^−6^) were retained as inputs for Weighted Gene Correlation Network Analysis (WGCNA),^42^ which produced a set of modules containing biologically related CpGs (Supplementary Fig S1). Standard settings for the WGCNA package in R software were used: unsigned network, minModulesize = 30, reassignment threshold = 0.05, and mergecutHeight = 0.25. Scale independence and mean connectivity assessment indicated a soft power threshold of 7 was optimal (Supplementary Fig S2). Each module is represented by its first principal component in further analyses.

#### DNAm Module Construction in EIMS

Because the EIMS analysis sample was smaller in number and DNAm was profiled using the Illumina 450 K array (providing only 64% as many CpGs as the EPIC 850 K array used in Ausimmune), we used sparse Partial Least Squares Discriminant Analysis (sPLS‐DA),^43^ a fully supervised machine‐learning approach better powered to derive MS‐associated DNAm modules in sparser data like that used in EIMS. The sPLS‐DA model was trained with a 75%/25% train/test split. Parameter tuning indicated the optimal model would include 3 components containing 1,000, 400, and 200 CpGs, respectively. After training, the components’ analysis indicated good discrimination between cases and controls.

#### Evaluations of DNAm Modules' Associations With MS Onset and With Risk Factors

We identified DNAm modules significantly associated with MS risk. Within each study, DNAm modules were ranked by the statistical significance of their associations with MS risk, ranging A1‐ to A5‐modules in Ausimmune and E1‐ and E2‐module in EIMS. We then evaluated the key risk factors, including EBV indices (IM history and anti‐EBNA IgG titer), lower serum 25(OH)D, low sun exposure, and HLA‐DRB1*1501 with these DNAm modules. We conducted a control‐only assessment of risk factors and DNAm modules in the larger Ausimmune Study. The smaller EIMS study (139 controls) was not suitable for this, particularly after risk stratification by HLA‐DRB1*1501 genotype.

#### Mediation Analyses of Exposure‐MS Associations and of MS‐Exposure Associations by DNAm Modules

DNAm modules were evaluated as mediators of exposure‐MS associations by counterfactual mediation analysis (*medflex* R package).^38^ This gave estimations of total (exposure‐outcome), direct (exposure‐to‐outcome independent of DNAm module), and indirect (exposure‐to‐outcome via DNA module) effects, as well as their statistical significances. We also report the proportion of total effect in exposure‐MS associations acting via the DNAm module as the proportion mediated (indirect/total effect ratio, as percent).

To support temporal ordering of the exposure‐mediator outcome measures here, as required for mediation analysis,^19^ we undertook the following analyses. We examined the extent that the time interval between FCD and interview was related to the measures, an approach we have used previously.^27^ We restricted to FDEs with no history of suspected demyelination (Supplementary Table S6). We tested whether the A1 and A2 DNAm modules were enriched for MS disease risk genes using genome‐wide association study (GWAS) metadata from the GWAS Catalog (https://www.ebi.ac.uk/gwas/). We assessed whether reverse‐pathway mediation analyses (case status to mediator to exposure) showed comparable magnitude and significance of mediation as seen in the primary (exposure to mediator to outcome) mediation analyses. As HLA‐DRB1*1501 is determined at conception, this was not assessed for potential reverse causation. In addition, to account for potential selection bias between those with and without DNAm data in Ausimmune, we undertook inverse probability weighting (IPW) methods, accounting for age, sex, region, education, and ancestry. Additional analyses included limiting EIMS analyses to those cases diagnosed in the preceding 5 years (n = 58), and, in both studies, analyses were done limiting to cases not on DMTs at the time of study (Ausimmune n = 163 and EIMS n = 52).

#### Using Gene Enrichment Analysis to Understand DNAm Module Functional Relevance

For each of the 4 DNAm modules found to be mediators of risk factor‐MS onset associations in Ausimmune and EIMS, the CpGs were mapped to their nearest gene and the gene‐set of each module was ordered by *p* values of correlations with their module. Each gene‐set was run through the pathfindR algorithm in R software, which performs active sub‐network enrichment analysis, with Reactome and Gene Ontology as reference pathway databases with Benjamini‐Hochberg adjustment for multiple comparisons. We assessed these results (i) for each module individually, and (ii) for the pathways that overlap with the Ausimmune modules’ results (given their enhanced biological information). To determine whether epigenetic factors had shared biological processes across the 2 studies, we used the hypergeometric test to calculate a fold enrichment and *p* value for the sets of pathways overlapping between these factors.^44^

#### Upstream Transcription Factors and DNAm Modules

We used the Lisa platform^45^ to infer the upstream regulators of the Ausimmune DNAm modules in silico, selecting the top 20 transcription factors (TFs) for each for further investigation. Using the Open Targets platform,^46^ we cross‐referenced each TF for association with MS, key MS treatments, and biological elements related to key MS‐associated transcription factors.

#### Reporting

We have ensured our analyses align with the STROBE^47^ reporting guidelines.

## Results

### Study Characteristics

The Ausimmune analyses compared 206 FCD cases who had predominantly converted to MS by the 10‐year follow‐up with 348 matched controls (Supplementary Table S1). The sample was 78% female subjects with a mean age 39.6 years. A minority of cases (20.9%) were using DMTs. Vitamin D supplement use was infrequent in this sample (1.8% of cases and 0.6% of controls). There were no material differences in the characteristics of cases or controls used in this project from those in the initial study (*p* ≥ 0.09). The time interval between FCD and interview did not relate to anti‐EBNA IgG titer and serum 25(OH)D (< 50 nmol/L).^27^

The EIMS sample with DNAm data was a matched subsample of the larger EIMS study (n = 140 MS cases and n = 139 matched controls) was 72.5% female subjects, with a mean age of 34.6 years. Two‐thirds of the cases were using DMTs at the time of blood sampling. Other study characteristics are shown in Supplementary Table S2.

### EBV Indices, Lower Vitamin D, Low Sun Exposure, and HLA‐DRB1*1501 Were Individually and Jointly Associated With MS Risk in Ausimmune and EIMS

Consistent with prior work,^27^, ^29^, ^48^ IM history, anti‐EBNA IgG titer, lower seasonally adjusted serum 25(OH)D (< 50 nmol/L), low pre‐onset sun exposure, and HLA‐DRB1*1501 were each associated with MS risk in the Ausimmune study (see Supplementary Table S1). Similarly, in the EIMS analysis subset, high anti‐EBNA IgG titer, low sun exposure, and HLA‐DRB1*1501 were associated with MS risk (see Supplementary Table S2), in keeping with previously published results.^6^, ^31^

**FIGURE 2:**
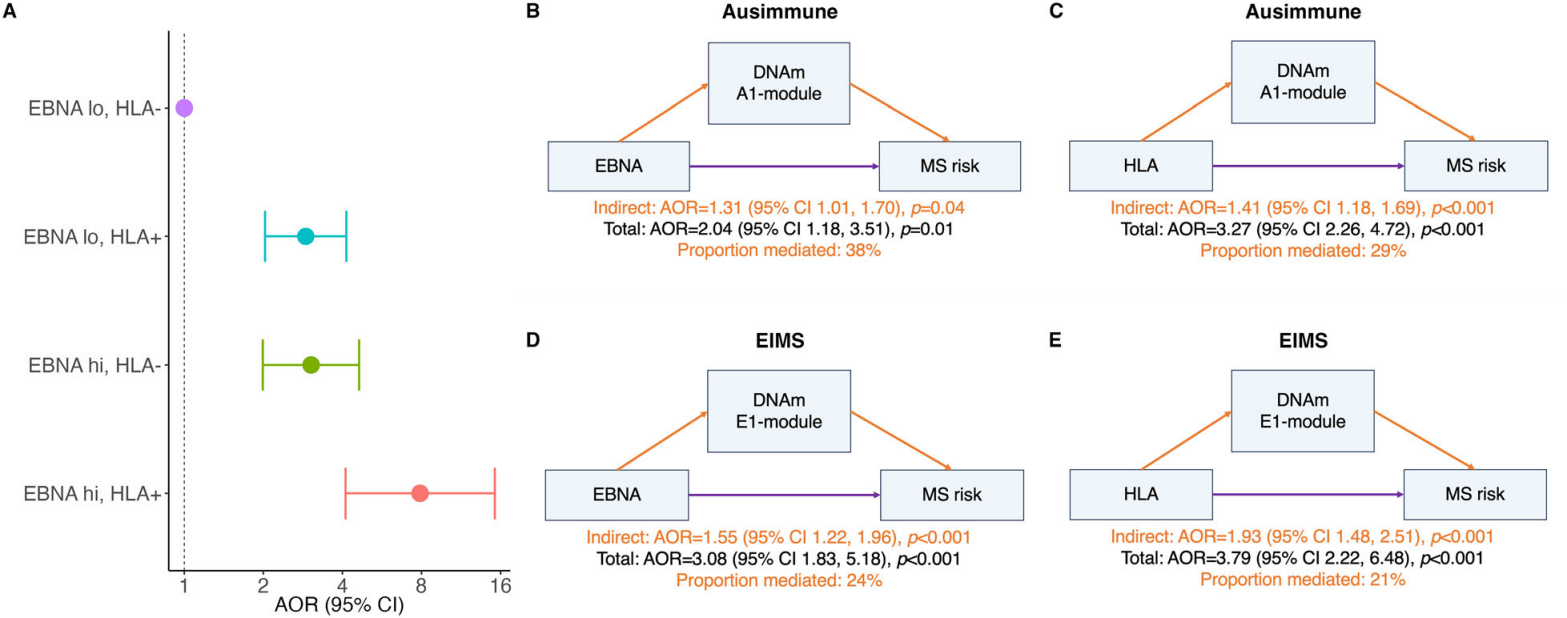
Elevated EBNA levels and HLA‐DRB1*1501 risk variant operate through common epigenetic DNAm modules to drive, in part, synergistic gene–environment interaction. (A) Synergistic interaction between anti‐EBNA IgG (EBNA) and HLA‐DRB1*1501 is evident in the meta‐analysis by Jacobs et al,^7^ which reported measures of interaction as follows: AP = 0.48 (95% CI = 0.26–0.71) and relative excess risk due to interaction (RERI) = 3.84 (95% CI: 1.19–6.49). EBNA lo and EBNA hi denote dichotomized anti‐EBNA IgG, dichotomized as top quartile (titer = 640 or 2,560) versus lower quartiles. HLA– and HLA+ represent HLA‐DRB1*1501 genotype (rs3135388) as AA/AG vs GG (risk). (B–E) mediation results demonstrate significant indirect effects for both anti‐EBNA IgG and HLA‐DRB1*1501 across both studies. In Ausimmune EBNA = anti‐EBNA IgG titer (top quartile is 640 and 2,560 titers vs rest) and HLA‐DRB1*1501 = HLA‐DRB1*1501 risk variant (rs3135388) is AA/AG versus GG (risk). In EIMS EBNA = anti‐EBNA IgG peptide, dichotomized at median (6,716) and HLA‐DRB1*1501 = HLA‐DRB1*1501 risk variant (rs3135388) is AA/AG versus GG (risk). AP = attributable proportion; 95% CI = 95% confidence interval; DNAm = DNA methylation; EBNA = Epstein Barr Nuclear Antigen; EIMS = Epidemiologic Investigations of Multiple Sclerosis; RERI = relative excess risk due to interaction. [Color figure can be viewed at www.annalsofneurology.org]

In both the Ausimmune and EIMS studies, we have previously reported interactions between HLA‐DRB1*1501 and anti‐EBNA IgG titer.^31^, ^48^ A recent meta‐analysis that included these studies demonstrated a supra‐additive interaction between these 2 factors (Fig 2A).^7^ Recently, Hedström et al reported positive additive patterns between anti‐EBV‐EBNA IgG titer and low sun exposure in EIMS.^6^ Using the Ausimmune data, we evaluated additive interactions between EBV indices and vitamin D/sun exposure. We found a significant positive additive interaction between anti‐EBNA IgG titer and both summer and winter sun exposure, those with both risk factors having a nearly 4‐fold greater MS risk compared to those with neither factor (Supplementary Table S3). Similar effects were observed using low seasonally adjusted serum 25(OH)D instead of low sun exposure, and IM history instead of anti‐EBNA IgG titer. Interaction was also evident on the multiplicative scale with lower 25(OH)D levels potentiating the adverse effects of EBV; in both studies, a doubling of anti‐EBNA IgG titer and lower serum 25(OH)D levels were found (Ausimmune: *p* = 0.022 and EIMS: *p* = 1.44 × 10^−8^; see Supplementary Fig S3).

Having now established the shared key risk factors and joint effects in these studies, we evaluated DNAm modules and related mediation using Ausimmune as the principal study and replication in the EIMS data, where possible.

### DNAm Modules Were Associated With Both MS Risk and with Key Risk Factors in Both Studies

In the Ausimmune study, all 5 of the DNAm modules were significantly associated with MS risk, whereas in the EIMS study, only the first 2 components were significantly associated with MS (all *p* < 0.001; Supplementary Table S4). The time interval between FCD and interview was not associated with any of the 5 modules in the Ausimmune study. These associations persisted in sensitivity analyses restricted to FDE cases in Ausimmune, as well as excluding all DMT‐treated cases in Ausimmune and EIMS.

**FIGURE 3:**
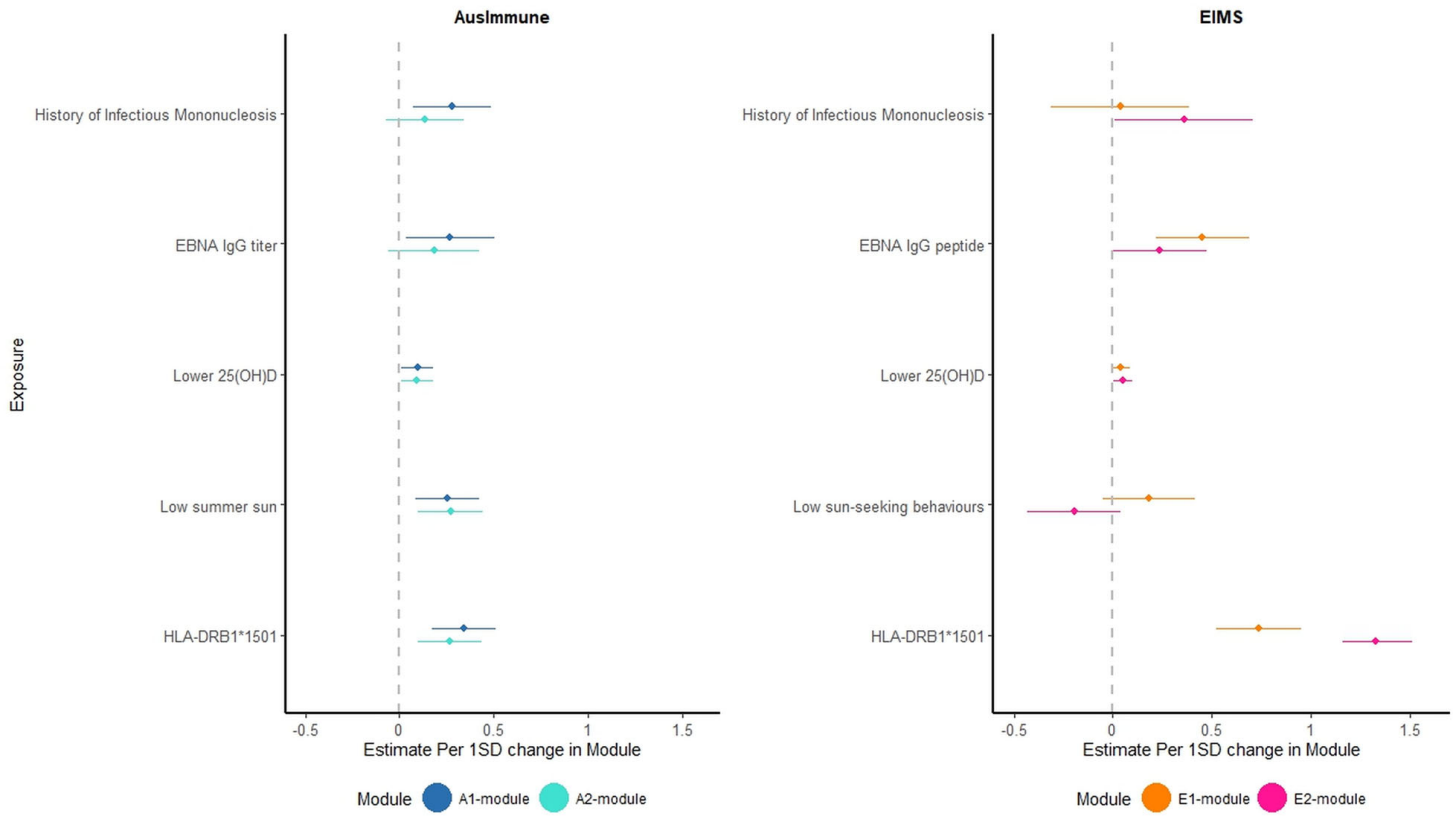
Key risk factors are associated with DNAm modules: the Ausimmune and EIMS studies. The compositions of these DNAm modules are described in the text. In the Ausimmune study, EBNA IgG titer = anti‐EBNA IgG titer (top quartile is 640 and 2,560 titers vs rest). In the EIMS study, EBNA IgG peptide = anti‐EBNA IgG peptide, dichotomized at median (6716). In both the Ausimmune and EIMS studies (cases and controls), lower 25(OH)D is continuous deseasonalized 25‐hydroxyvitamin D (25(OH)D), reversed to be in consistent positive association as other covariates in figure, expressed in units of 10 nmol/L. In Ausimmune, low summer sun was defined from self‐reported sun exposure less than 2 hours/day in summer or winter in 3 years preceding FCD. Low sun exposure in EIMS was defined from responses to 3 self‐reported questions regarding sun exposure (frequency of sunbathing, frequency of recent travel to a sunnier country than Sweden, and frequency of use of sunbeds), estimating a composite score ranging from 3 to 12, here, dichotomized and low sun exposure defined as sun score ≤ 6. In both the Ausimmune and EIMS studies, HLA‐DRB1*1501 = HLA‐DRB1*1501 risk variant (rs3135388) is AA/AG vs GG (risk). For control‐only results, please see text. DNAm = DNA methylation; EBNA = Epstein Barr Nuclear Antigen; EIMS = Epidemiologic Investigations of Multiple Sclerosis; FCD = first clinical diagnosis. [Color figure can be viewed at www.annalsofneurology.org]

Evaluating MS risk‐associated DNAm modules’ associations with the key exposures in each study, including higher anti‐EBNA IgG titer, lower serum 25(OH)D levels, and HLA‐DRB1*1501 (Fig 3 and Supplementary Table S5), only the A1 and A2 modules in Ausimmune were consistently associated with EBV indices or sun/vitamin D parameters. Therefore, these 2 top ranked modules were examined further but A3 to A5 were not.

Among the Ausimmune controls, the exposure‐DNAm module associations were still evident, some varying by HLA‐DRB1*1501. For example, higher anti‐EBNA IgG titers were more strongly associated with the A1‐module among those with HLA‐DRB1*1501 compared with those without (adjusted mean difference [AMD] = 0.02, 95% confidence interval [CI] = 0.00 to 0.04 vs. −0.05, 95% CI = −0.02 to –0.13), p_difference_ = 0.05). For the A2‐module, higher 25(OH)D levels were inversely associated with module scores only among those with HLA‐DRB1*1501 (β = −0.0038, 95% CI = −0.0062 to 0.0014, p_difference_ = 0.009). These restricted analyses indicate that even among those without MS, environmental factors are associated with the 2 key DNAm modules in Ausimmune and HLA‐DRB1*1501 status is a modifying element.

### DNAm Modules Have Enrichment for GWAS MS Risk Genes to Strengthen Evidence on the Direction of Causation

Assessing gene‐set enrichment for GWAS MS risk genes in the A1‐ and A2‐modules, we found that both modules showed significant over‐representation, with particularly strong enrichment for the highest confidence MS loci among the most connected genes in each network. The top 200 genes in the A1‐module contained 3.5 times more of the top 200 MS GWAS hits than expected by chance (hypergeometric *p* = 0.004), whereas the A2‐module contained 3.0 times more (*p* = 0.015). Because these genes are genetically linked to MS, they are causally prior, supporting the interpretation that the associated epigenetic changes are upstream of clinical onset and represent a cause rather than a consequence of MS. This enrichment was robust across essentially all cutoffs for the A1‐ and A2‐modules and MS GWAS gene sets (data not shown).

### DNAm Modules Mediated Multiple Risk Factor Associations With MS Risk in Ausimmune and EIMS

In the Ausimmune study, both DNAm modules mediated multiple exposure‐MS onset associations. The A1‐module significantly mediated associations of anti‐EBNA IgG titer (38%, *p* = 0.040; see the Table 1 and Fig 2B), lower serum 25(OH)D (44%, *p* = 0.037), and HLA‐DRB1*1501 (29%, *p* < 0.001; Fig 2C) with MS risk. The A2‐module mediated the associations of lower serum 25(OH)D (38%, *p* = 0.001), low summer sun exposure (47%, *p* = 0.002), and HLA‐DRB1*1501 (21%, *p* = 0.002) with MS risk. These results persisted on restriction to FDE cases (see Supplementary Table S6) and after excluding DMT‐treated cases (Supplementary Table S7), and applying IPW (data not shown).

**TABLE 1:** The Ausimmune Study: Mediation by DNAm Modules of the Association Between Key Risk Factors and MS Onset

	Total Effect		Direct Effect		Indirect Effect		Percent Indirect Effect
	OR (95% CI)	*p*	OR (95% CI)	*p*	OR (95% CI)	*p*	
A1‐module							
History of infectious mononucleosis	**2.41 (1.55, 3.74)**	**< 0.001**	**1.73 (1.19, 2.52)**	**0.004**	**1.39 (1.1, 1.75)**	**0.005**	**37.0%**
Anti‐EBNA IgG titer^a^	**2.04 (1.18, 3.51)**	**0.011**	1.55 (0.95, 2.53)	0.079	**1.31 (1.01, 1.7)**	**0.040**	**38.0%**
Low 25(OH)D^b^	**1.08 (1.02, 1.16)**	**0.012**	1.05 (0.99, 1.1)	0.092	**1.04 (1.00, 1.07)**	**0.037**	**44.0%**
Low summer sun^c^	**1.73 (1.22, 2.46)**	**0.002**	1.30 (0.95, 1.78)	0.095	**1.33 (1.10, 1.61)**	**0.003**	**51.0%**
HLA‐DRB1*1501 ^d^	**3.27 (2.26, 4.72)**	**< 0.001**	**2.32 (1.69, 3.18)**	**< 0.001**	**1.41 (1.18, 1.69)**	**< 0.001**	**29.0%**
A2‐module							
History of Infectious Mononucleosis	**2.45 (1.57, 3.82)**	**< 0.001**	**2.08 (1.39, 3.11)**	**< 0.001**	1.18 (0.97, 1.43)	0.10	18.0%
Anti‐EBNA IgG titer^a^	**2.01 (1.18, 3.42)**	**0.010**	**1.70 (1.04, 2.77)**	**0.033**	1.18 (0.96, 1.46)	0.12	24.0%
Low 25(OH)D^b^	**1.08 (1.02, 1.15)**	**0.011**	1.05 (0.99, 1.11)	0.13	**1.04 (1.01, 1.06)**	**0.013**	**38.0%**
Low summer sun^c^	**1.72 (1.22, 2.44)**	**0.002**	1.33 (0.96, 1.85)	0.085	**1.29 (1.1, 1.52)**	**0.002**	**47.0%**
HLA‐DRB1*1501 ^d^	**3.27 (2.27, 4.73)**	**< 0.001**	**2.54 (1.82, 3.54)**	**< 0.001**	**1.29 (1.1, 1.51)**	**0.002**	**21.0%**

Analyses using the medflex package in R software, outcome model using logistic regression, mediator model using linear regression, measures of association from main exposure‐outcome mediation model presented as aOR (95% CI), adjusted for age, sex, and study region. Results in boldface denote statistical significance (*p* < 0.05).

^a^ Anti‐EBNA IgG titer (top quartile is 640 and 2,560 titers vs rest).

^b^ The 25(OH)D is seasonally adjusted, per 10 nmol/L, reversed for direction of MS risk.

^c^ Low sun exposure in Ausimmune is defined from self‐reported sun exposure less than 2 hours/day in summer or winter in 3 years preceding FCD.

^d^ HLA‐DRB1*1501 risk genotype (rs3135388) is AA/AG vs GG (risk).

25(OH)D = 25‐hydroxyvitamin D; aOR = adjusted odds ratio; 95% CI = 95% confidence interval; DNAm = DNA methylation; EBNA = Epstein Barr Nuclear Antigen; FCD = first clinical diagnosis; HLA = Human Leukocyte Antigen; MS = multiple sclerosis; OR = odds ratio.

In the EIMS study, the E1‐module mediated the associations of the following with MS: anti‐EBNA IgG titer (39%, *p* < 0.001; Fig 2D) and HLA‐DRB1*1501 (49%, *p* < 0.001; Fig 2E), as well as showing a near‐significant trend for lower serum 25(OH)D (25%, *p* = 0.077; Supplementary Table S8). The E2‐module mediated the associations of the following with MS: lower 25(OH)D (25%, *p* = 0.016) and HLA‐DRB1*1501 (53%, *p* = 0.003). On excluding DMT‐treated cases (n = 87, 62.1%), all these indirect effects persisted in magnitude with minimal attenuation (Supplementary Table S9). Further, all indirect effects persisted when restricted to the cases who were ≤ 5 years post‐diagnosis (Supplementary Table S10).

To assess potential reverse causation, we examined if MS‐to‐environmental factor associations could be mediated by DNAm modules. In neither the Ausimmune nor EIMS study, no mediation in this reverse direction reaching the 5% significance threshold was seen for any associations (Supplementary Tables S11 and S12).

### Pathways Enriched for DNAm Modules Involve Key MS‐Relevant Biological Pathways

In the Ausimmune study, the A1‐module included 436 CpGs across 407 genes. The most enriched Reactome pathway of this module's gene‐set was “SCF‐KIT signaling”. Other key enriched pathways involved cell‐to‐cell signaling, cellular stress response, NOTCH1 signaling, phagocytosis, Leishmaniasis infection, and signal transduction.

The A2‐module included 687 CpGs across 627 genes and its gene‐set was most enriched for the “SUMO E3 ligates SUMOylate target proteins” Reactome pathway. Other key enriched Reactome pathways relate to SUMOylation, signal transduction, and infection response.

In the EIMS study, the E1‐module included 1,000 CpGs across 795 genes and accounted for 78.4% (*p* < 0.001) of the DNAm‐MS covariance in EIMS. Key enriched Reactome pathways for this module's gene‐set included “RAC1 GTPase cycle”, “SUMOylation”, “Platelet activation and aggregation”, “Signal transduction”, “Leishmania phagocytosis”, “Cell‐cell signaling”, and “Transcription regulation”.

The E2‐module included 400 CpGs across 300 genes and accounted for 18% of the DNAm‐MS covariance in EIMS. Other key enriched Reactome pathways included “CD3 and T‐cell receptor signaling”, “Interferon signaling”, “MHC I antigen presentation”, “Cellular mitosis”, and “Transcription regulation”.

### DNAm Module Pathway Enrichment Results Were Consistent Across Ausimmune and EIMS

**FIGURE 4:**
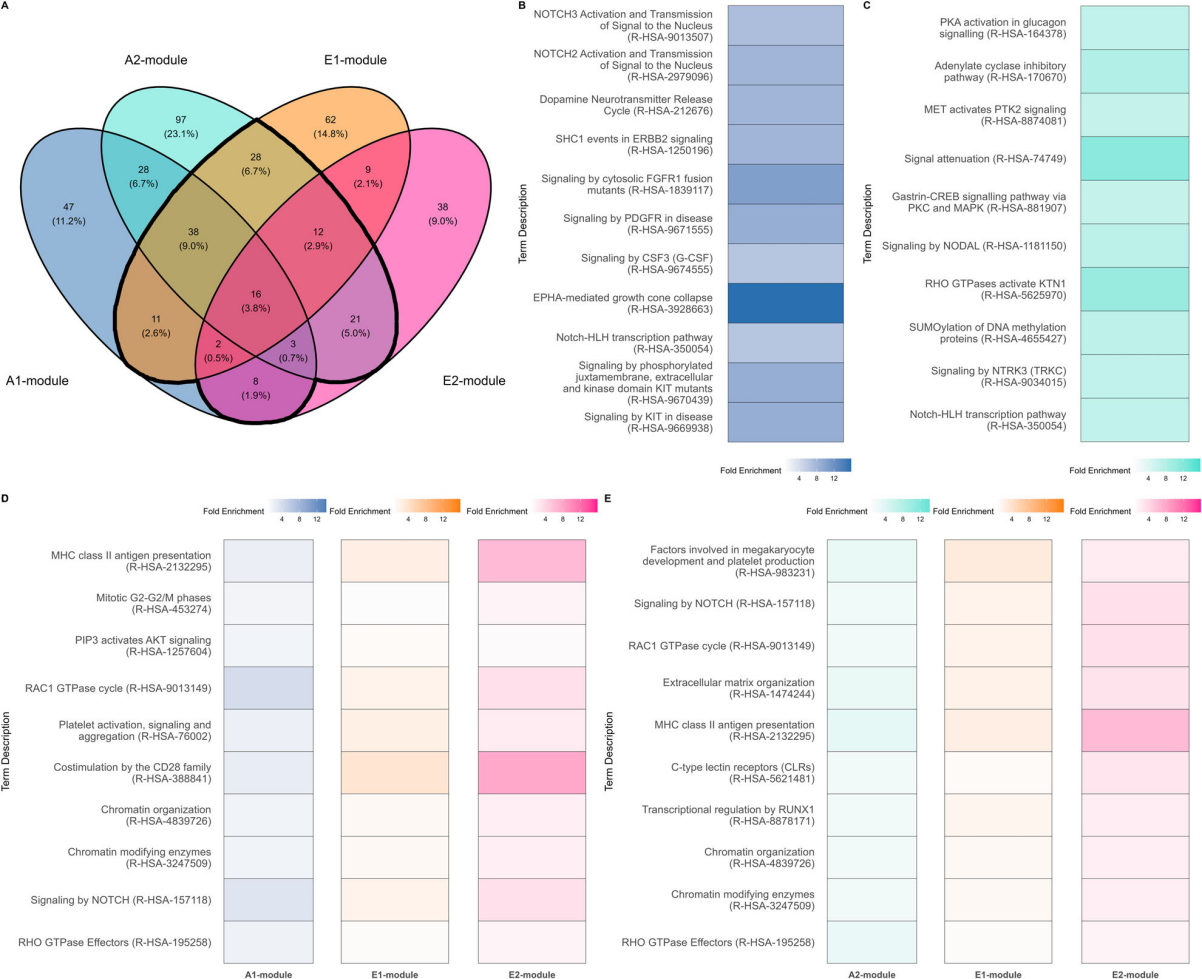
Pathway visualization across the Ausimmune and EIMS DNAm modules. (A) Venn diagram showing the overlap of enriched pathways for each DNAm module. Total number of enriched pathways: 559 (311 in Ausimmune and 248 in EIMS). Pathways enriched in both Ausimmune modules that are also enriched in EIMS: 57 of 85 (67.1%). Enriched pathways from either Ausimmune module also enriched in EIMS networks: 139 of 311 (44.7%). (B) Fold enrichment for the top 10 enriched pathways for the A1‐module. (C) Fold enrichment for the top 10 enriched pathways for the A2‐module. (D) Top 10 enriched terms in the A1‐module that also appear in both EIMS DNAm modules. (E) Top 10 enriched terms in the A2‐module that also appear in both EIMS DNAm modules. DNAm = DNA methylation; EIMS = Epidemiologic Investigations of Multiple Sclerosis. [Color figure can be viewed at www.annalsofneurology.org]

Substantial cross‐study overlaps were evident for the enriched pathway set identified within the DNAm modules. Of the Reactome pathways enriched in either Ausimmune DNAm module, nearly half (44.7%, 139/311) were also enriched in at least one of the EIMS modules. This finding is 4.82‐fold higher than chance (*p* = 8.48 × 10^−76^). Of those enriched in both Ausimmune DNAm modules, 67.1% (57/85) were also enriched in at least one EIMS module. This is 7.23‐fold higher than chance (*p* = 6.59 × 10^−41^; Figure 4). Of the pathways enriched in the gene‐sets of all 4 DNAm modules considered, the most enriched terms were “Interferon alpha/beta signaling” and “Interferon signaling” (Supplementary Table S13). Using the Gene Ontology database as an alternative reference, 5 terms were enriched across the DNAm modules in both studies (Supplementary Table S14). A total of 21 pathways were enriched across both the Ausimmune and EIMS studies. Several of these pathways are of particularly direct interest to MS, including “Signaling by B‐cell Receptor”, “Interferon alpha/beta”, and “Interferon signaling”.

### Inferred Upstream Transcription Factors for the Ausimmune DNA Modules

We examined higher‐order functional coordination by upstream regulators. In Open Targets,^46^ we examined the top 20 transcription factors (TFs, determined by the LISA platform^45^) for each DNAm module in Ausimmune. From this, we observed that 45% (n = 9) of TFs linked to the A1‐module and 35% (n = 7) of TFs linked to the A2‐module had been previously annotated to MS.^46^ TFs associated with interferon‐beta‐1A, IFNAR1, and IFNAR2 are related to 2 key TFs associated with MS in the A2‐module's gene‐set, STAT4 and interferon regulatory factor 5 (IRF5).^46^

## Discussion

Here, we demonstrated that the associations of key risk factors for MS, including EBV indices (infectious mononucleosis history and higher anti‐EBNA IgG titer), act individually and jointly with other risk factors, including lower serum 25(OH)D, low sun exposure, and HLA‐DRB1*1501. Importantly, we demonstrate that these risk factors act upon MS risk through common differential DNAm in genes in pathways relevant to immune and neurological function. Compelling evidence recently showed that EBV infection is a causal risk factor for MS.^49^ However, as Hedström et al recently stated, “while EBV infection appears to be a cause for MS, it must work with other co‐factors to provide sufficient cause.”^50^ Our findings add clarity to this, showing: (i) EBV indices act in concert with other risk factors – lower vitamin D levels, low sun exposure, and HLA‐DRB1*1501 risk variant to initiate disease, and (ii) EBV and these other factors act via common epigenetic programming, specifically differential DNA methylation to provide sufficient cause for MS onset.

Prior work in the Ausimmune and EIMS studies has shown significant differences in DNAm profiles between MS onset cases and controls.^22^ Here, we show that the effects of EBV and other key risk factors on MS risk in each study are mediated by common DNAm modules containing pathways involved in immune and neurological function. These DNAm module scores mediated 18 to 38% and 39% of the associations between EBV and MS risk in Ausimmune and EIMS, respectively. Further, 38 to 51% and 25% of the associations of lower 25(OH)D levels and low pre‐onset sun exposure with MS risk were mediated by DNAm modules in Ausimmune and EIMS, respectively. Finally, 21 to 29% and 49 to 53% of HLA‐DRB1*1501 associations with MS risk were mediated by DNAm modules in Ausimmune and EIMS, respectively. This indicates that multiple co‐factors for MS risk can be mediated by shared epigenetic modules.

Overall, the findings are consistent with the Rothman framework that risk factor interaction indicates factors that act as component causes in sufficient cause pathway.^5^ Dwyer et al articulated this further: having identified one component cause in the risk structure leading to disease, other risk factors for the disease outcome become more discernible, particularly if they act along the same pathway to disease.^51^ Here, we provide strong evidence showing that the previously demonstrated gene–environment interactions between HLA‐DRB1*1501 and higher anti‐EBV IgG titer^7^ represents, in part, that both factors act through epigenetic alteration of pathways involving MHC class II, NOTCH, and GTPase signaling. HLA‐DRB1*1501 partly acts in MS by priming hyper‐reactive T‐cell compartments, which then control EBV infection/reactivation less efficiently, as well as potentially including T‐cell variants that are cross‐reactive against host antigens.^52^ In addition, our findings in Ausimmmune of interactions between lower vitamin D levels or low sun exposure and EBV indices, supporting previous findings in EIMS,^6^ informs the interpretation of the mediation by the same DNAm modules (A1 and A2), particularly in loci in pathways involving interferon signaling, NOTCH, and RUNX1 as relevant for these risk factors also.

Evaluating mediating DNAm module composition, biologically plausible pathways were enriched in gene‐sets in both the Ausimmune and EIMS studies. The Reactome pathways most enriched for the Ausimmune A1‐module (“RAC1 GTPase cycle” and “Signaling by NOTCH”) were highly ranked in enriched pathways for the A2‐module module and for both the EIMS DNAm modules. These 2 pathways are involved in the regulation of cell function, proliferation, and differentiation in immune cells and in the CNS,^53^, ^54^ and both interact with EBV infection and low vitamin D status.^55^, ^56^ In EIMS, the most enriched Reactome pathways for the E1‐module and the E2‐module gene‐sets were “Interferon alpha/beta signaling” and “Interferon signaling”, which were also significant for Ausimmune after false discovery rate (FDR) correction. Interferon signaling has clear relevance in MS, as a fundamental element of innate and adaptive immune response pathways,^57^ and interacts with EBV indices^58^ and low vitamin D status,^59^ among other factors.

Substantial cross‐study overlap of pathways enriched for DNAm module gene‐sets was evident. Of Reactome pathways enriched in Ausimmune DNAm module gene‐sets, 45% were also enriched in EIMS, this more than 4‐fold enrichment significantly more than would be expected by chance. Further, analysis of inferred upstream transcription factors of the Ausimmune DNAm module gene‐sets showed almost half of the top 20 TFs for each module had been a priori annotated to MS. Of further interest were the links among the interferon‐beta‐associated TFs, IFNAR1 and IFNAR2, and key TFs for the A2‐module, STAT4, and IRF5, via the JAK–STAT pathway and related to type‐I interferons, respectively. IFNAR1 and IFNAR2 form the receptor for type‐I interferons and are essential for activating the JAK–STAT signaling pathway which regulates transcription of interferon‐stimulated genes,^46^ whereas STAT4 and IRF5 have links to IFNAR1 and 2 via the JAK–STAT pathway and type‐I interferons, respectively. Our results may also inform the female preponderance in MS: AR, the top TF in the A1‐module, is a steroid hormone receptor activated by androgen binding and is currently in trials for MS treatment. These pathways implicated here thus have links not only to EBV indices and low vitamin D as single risk factors but also to the interaction between them (“RAC1 GTPase cycle”, Signaling by NOTCH”, and “Interferon signaling”). In summary, here, we find strong in silico links from DNAm modules to MS, MS‐related pathways, and treatments.

Strengths of this work include the comprehensive data on environmental, genetic, and DNAm measures available in these 2 MS case‐control studies. This enabled us to identify mediation by DNAm modules in Ausimmune and then replicating these findings using similar DNAm modules in EIMS. Recent advances in highly dimensioned data analysis allowed us to investigate multifactorial causation more deeply than previous work. Although the studies were of moderate size, clear patterns were evident. Statistical power was enhanced using network‐based methods rather than individual CpGs and reduced the burden of extensive agnostic false discovery adjustments for the latter.^60^

These findings were consistent across the 2 studies, despite differences in the DNAm arrays used and associated module formation methods. That is replication was evident at 4 key levels across the 2 studies: (i) replication of the exposure‐outcome associations was obtained; (ii) replication was obtained at the level of interaction effects; and (iii) identification of mediation effects in both studies strengthens the evidence for underlying epigenetic mechanisms. The DNAm modules in the Ausimmune and EIMS studies were significant mediators of the same environmental and genetic MS risk factors; (iv) pathway enrichment analyses of the top mediators from each cohort revealed overlapping biological pathways, suggesting convergent molecular mechanisms. Nearly half of the enriched pathways detected in Ausimmune DNAm modules were also found in the DNAm modules generated in EIMS.

Whole‐blood DNAm signatures, as measured here, have been associated with clinical phenotype in MS^15^ and are also relevant to the CNS, disordered DNAm in both of these have been shown in MS.^9^, ^14^ Our analyses controlled for the estimated cell‐type proportions in each sample using cell deconvolution, as described.^40^ In so doing, we can interpret results consistently between samples, despite any inter‐sample differences in cell‐type fractions. Of note, however, we did not evaluate cell type‐specific DNAm effects here, and further work exploring DNAm profiles within specific cell populations would be worthwhile. Although the use of DNAm modules was informative, it did not allow us to examine the potential mediating roles of individual CpGs. Although high‐dimensional approaches have been developed to do this,^61^ individual CpG effects are usually small in magnitude and thus they require larger studies than those examined here.

A strength of these well‐known case‐control studies is that controls were selected from population‐based national registries. Nevertheless, selection bias is a concern. The samples used in this project were smaller, and the EIMS subsample used heavy matching to balance case and control characteristics. In Ausimmune, the participants did not differ materially from the initial study population. We conducted several sensitivity analyses. First, the mediation findings persisted when restricting cases to FDE (in Ausimmune) or early post‐diagnosis (in EIMS). We also compared forward (risk factor‐to‐MS) to reverse (MS‐to‐risk factor) mediation, showing that reverse pathway mediation was lower in magnitude and not significant at the 5% level. The DNAm module associations were evident for samples restricted to treatment‐naïve in both studies and FDEs in Ausimmune. Control analyses indicate that even among those without MS, environmental factors were associated with the 2 key DNAm modules in Ausimmune and HLA‐DRB1*1501 risk status is a modulating element. The latter biological feature is over‐represented in MS cases and may have contributed to the mediation findings observed.^60^

Vanderweele and colleagues^19^, ^62^ have described the fundamental importance of temporal ordering of exposure, mediator, and outcome in mediation analyses. In many studies, this is not straightforward; almost half of observational studies assessed in a 2021 scoping mediation review were cross‐sectional or case‐control in design.^63^ Such study designs require further consideration evaluating the temporal ordering required for mediation analysis. Therefore, we undertook several supporting and sensitivity analyses. Including showing exposure‐DNAm associations among controls, and supporting temporal ordering of exposure‐mediator‐outcome and mediator‐outcome measures using restriction to FDEs with no history of suspected demyelination and assessing case‐only time‐since onset durations for exposure and mediator measures. In addition, in showing that MS risk‐associated genes were enriched within the DNAm modules via genomic concordance analysis, we can further support the DNAm to MS directionality.^25^, ^64^ The significant overlap seen for these DNAm modules supports that epigenetic disruption in each module is a cause for rather than a consequence of MS. Finally, in undertaking reverse pathway mediation analyses and quantitatively assessing the MS‐mediator‐exposure mediation pathways and comparing with the primary exposure‐mediator‐MS mediation pathways, we could directly assess both the potential for reverse causality as an explanation for the observed associations. Mediation was not evident in reverse‐mediation analyses, making reverse causality an unlikely explanation for the findings in our main analyses.

Nevertheless, substantiation of these findings in prospective cohort studies will be important, and we acknowledge that a prospective cohort study design is best placed to directly evaluate mediation. However, given the incidence of MS and its typical onset in adulthood, whereas the potential risk factors and their exposure periods occur across the life‐course, the feasibility of such prospective studies is limited. There is also a notable paucity of either case‐control or prospective cohort studies in people living with MS that comprehensively assess multiple risk factors, along with genome‐wide DNAm or other epigenetic parameters. We thus encourage the implementation of such approaches in future work.

Substantiation of these results in large prospective cohort studies, ideally with identical data structures to allow cross‐cohort replication in all aspects, would also be valuable for other analyses, including providing opportunities to investigate age and sex in a way that was not possible in these matched case‐control studies. Subject to such substantiation, identifying DNAm profiles associated with disease onset may guide the development of epigenetic‐based interventions, as well as highlighting associated cellular processes with potential for targeting by pharmacotherapy. The properties of treatments targeting DNAm offer an opportunity for novel MS therapies.^21^ DNAm inhibitors are already in active use in other clinical settings such as cancer,^65^, ^66^ so translation to application in MS could be feasible. We have recently demonstrated that interferon‐beta treatment recruits the endogenous anti‐viral molecular machinery through epigenetic alteration.^17^ Whether epigenetic programming affects other DMTs in MS requires investigation.

In conclusion, DNAm modules with common biological underpinnings significantly mediated the associations of canonical environmental and genetic risk factors for MS onset, independent of treatment effects. These findings substantiate the important roles of these multiple risk factors in the etiology of MS and indicate that they act, in part, through differential methylation of genes involved in relevant biological pathways. Pathways with evidence of cross‐study replication include interferon‐alpha/beta and B‐cell receptor signaling. Importantly, EBV infection and associated host response act upon MS risk via common epigenetic pathways that lower serum 25(OH)D, low sun exposure, and HLA‐DRB1*1501 risk variant share. These findings may inform risk stratification and future translation for epigenetic prognosis and intervention.

## Author Contributions

A.L.P., J.L.S., R.M.L., B.V.T., T.J.K., M.J., L.A., A.K.H., P.S., T.O., and The Ausimmune Investigators Group contributed to the conceptualization and design of the studies. S.S.Y., E.M., S.A.T., S.M.T., A.E., R.M.L., T.J.K., J.L.S., R.J.S., A.X., V.E.M., R.M.L., B.V.T., S.A.B., I.vD.M., M.W.M., B.A.L., B.N., R.Sa., R.Sc., A.K.H., P.S., T.O., M.J., L.A., A.L.P., and The Ausimmune Investigators Group contributed to the acquisition and analysis of data. S.S.Y., E.M., A.E., M.M., S.A.T., S.M.T., and A.L.P. contributed to drafting the text or preparing the figures.

## Potential Conflicts of Interest

Nothing to report.

## Supporting information


**Supplementary Data S1** Supporting Information.

**Supplementary Data S2** Ausimmune Investigators Group.

## Data Availability

The Ausimmune and AusLong data used in this paper are available under restricted access for participant privacy. Requests for data access should be directed to the corresponding author, Professor Anne‐Louise Ponsonby (annelouise.ponsonby@florey.edu.au). Requests will be considered on scientific and ethical grounds and, if approved, provided under collaborative research agreements.

## References

[1] Charabati M , Wheeler MA , Weiner HL , Quintana FJ . Multiple sclerosis: neuroimmune crosstalk and therapeutic targeting. Cell 2023;186:1309–1327. 37001498 10.1016/j.cell.2023.03.008 PMC10119687

[2] Almohmeed YH , Avenell A , Aucott L , Vickers MA . Systematic review and meta‐analysis of the sero‐epidemiological association between Epstein Barr virus and multiple sclerosis. PLoS One 2013;8:e61110. 23585874 10.1371/journal.pone.0061110 PMC3621759

[3] International Multiple Sclerosis Genetics C . Multiple sclerosis genomic map implicates peripheral immune cells and microglia in susceptibility. Science 2019;365(6460):eaav7188. 31604244 10.1126/science.aav7188 PMC7241648

[4] Vietzen H , Berger SM , Kuhner LM , et al. Ineffective control of Epstein‐Barr‐virus‐induced autoimmunity increases the risk for multiple sclerosis. Cell 2023;186:5705–5718.e13. 38091993 10.1016/j.cell.2023.11.015

[5] Rothman KJ , Greenland S , Poole C , Lash TL . Chapter 2: causation and causal inference. In: Rothman KJ , Greenland S , eds. Modern Epidemiology. 3rd ed. Philadelphia: Lippincott, Williams & Wilkins, 2008.

[6] Hedström AK , Huang J , Brenner N , et al. Low sun exposure acts synergistically with high Epstein‐Barr nuclear antigen 1 (EBNA‐1) antibody levels in multiple sclerosis etiology. Eur J Neurol 2021;28:4146–4152. 34435414 10.1111/ene.15082

[7] Jacobs BM , Giovannoni G , Cuzick J , Dobson R . Systematic review and meta‐analysis of the association between Epstein‐Barr virus, multiple sclerosis and other risk factors. Mult Scler 2020;26:1281–1297. 32202208 10.1177/1352458520907901 PMC7543008

[8] Olsson T , Barcellos LF , Alfredsson L . Interactions between genetic, lifestyle and environmental risk factors for multiple sclerosis. Nat Rev Neurol 2017;13:25–36. 27934854 10.1038/nrneurol.2016.187

[9] Chan VS . Epigenetics in multiple sclerosis. In: Chang C , Lu Q , eds. Epigenetics in Allergy and Autoimmunity Advances in Experimental Medicine and Biology. Singapore: Springer, 2020:309–374. 10.1007/978-981-15-3449-2_12 32445101

[10] Huynh JL , Casaccia P . Epigenetic mechanisms in multiple sclerosis: implications for pathogenesis and treatment. Lancet Neurol 2013;12:195–206. 23332363 10.1016/S1474-4422(12)70309-5 PMC3690378

[11] Maltby VE , Lea RA , Sanders KA , et al. Differential methylation at MHC in CD4(+) T cells is associated with multiple sclerosis independently of HLA‐DRB1. Clin Epigenetics 2017;9:71. 28729889 10.1186/s13148-017-0371-1 PMC5516341

[12] Ewing E , Kular L , Fernandes SJ , et al. Combining evidence from four immune cell types identifies DNA methylation patterns that implicate functionally distinct pathways during multiple sclerosis progression. EBioMedicine 2019;43:411–423. 31053557 10.1016/j.ebiom.2019.04.042 PMC6558224

[13] Maltby VE , Graves MC , Lea RA , et al. Genome‐wide DNA methylation profiling of CD8+ T cells shows a distinct epigenetic signature to CD4+ T cells in multiple sclerosis patients. Clin Epigenetics 2015;7:118. 26550040 10.1186/s13148-015-0152-7 PMC4635618

[14] Huynh JL , Garg P , Thin TH , et al. Epigenome‐wide differences in pathology‐free regions of multiple sclerosis‐affected brains. Nat Neurosci 2014;17:121–130. 24270187 10.1038/nn.3588 PMC3934491

[15] Campagna MP , Xavier A , Lea RA , et al. Whole‐blood methylation signatures are associated with and accurately classify multiple sclerosis disease severity. Clin Epigenetics 2022;14:194. 36585691 10.1186/s13148-022-01397-2 PMC9805090

[16] Pinto‐Medel MJ , Oliver‐Martos B , Urbaneja‐Romero P , et al. Global methylation correlates with clinical status in multiple sclerosis patients in the first year of IFNbeta treatment. Sci Rep 2017;7:8727. 28821874 10.1038/s41598-017-09301-2 PMC5562733

[17] Xavier A , Campagna MP , Maltby VE , et al. Interferon beta treatment is a potent and targeted epigenetic modifier in multiple sclerosis. Front Immunol 2023;14:1162796. 37325639 10.3389/fimmu.2023.1162796 PMC10266220

[18] Lucas R , Ponsonby AL , McMichael A , et al. Observational analytic studies in multiple sclerosis: controlling bias through study design and conduct. The Australian multicentre study of environment and immune function. Multiple Sclerosis (Houndmills, Basingstoke, England) 2007;13:827–839. 17881396 10.1177/1352458507077174

[19] VanderWeele TJ . Mediation analysis: a Practitioner's guide. Annu Rev Public Health 2016;37:17–32. 26653405 10.1146/annurev-publhealth-032315-021402

[20] Eslahi M , Nematbakhsh N , Dastmalchi N , et al. An updated review of epigenetic‐related mechanisms and their contribution to multiple sclerosis disease. CNS Neurol Disord Drug Targets 2022;22(3):381–393. 10.2174/1871527321666220119104649 35043771

[21] Kular L , Liu Y , Ruhrmann S , et al. DNA methylation as a mediator of HLA‐DRB1*15:01 and a protective variant in multiple sclerosis. Nat Commun 2018;9:2397. 29921915 10.1038/s41467-018-04732-5 PMC6008330

[22] Xavier A , Maltby VE , Ewing E , et al. DNA methylation signatures of multiple sclerosis occur independently of known genetic risk and are primarily attributed to B cells and monocytes. Int J Mol Sci 2023;24(16):12576. 37628757 10.3390/ijms241612576 PMC10454485

[23] Guo R , Gewurz BE . Epigenetic control of the Epstein‐Barr lifecycle. Curr Opin Virol 2022;52:78–88. 34891084 10.1016/j.coviro.2021.11.013 PMC9112224

[24] Meng W , Zhu Z , Jiang X , et al. DNA methylation mediates genotype and smoking interaction in the development of anti‐citrullinated peptide antibody‐positive rheumatoid arthritis. Arthritis Res Ther 2017;19:71. 28356135 10.1186/s13075-017-1276-2 PMC5372280

[25] Badam TVS , de Weerd HA , Martinez‐Enguita D , et al. A validated generally applicable approach using the systematic assessment of disease modules by GWAS reveals a multi‐omic module strongly associated with risk factors in multiple sclerosis. BMC Genomics 2021;22:631. 34461822 10.1186/s12864-021-07935-1 PMC8404328

[26] Sahoo K , Sundararajan V . Methods in DNA methylation array dataset analysis: a review. Comput Struct Biotechnol J 2024;23:2304–2325. 38845821 10.1016/j.csbj.2024.05.015 PMC11153885

[27] Lucas RM , Ponsonby AL , Dear K , et al. Sun exposure and vitamin D are independent risk factors for CNS demyelination. Neurology 2011;76:540–548. 21300969 10.1212/WNL.0b013e31820af93d

[28] Polman CH , Reingold SC , Banwell B , et al. Diagnostic criteria for multiple sclerosis: 2010 revisions to the McDonald criteria. Ann Neurol 2011;69:292–302. 21387374 10.1002/ana.22366 PMC3084507

[29] Lucas RM , Ponsonby AL , Dear K , et al. Current and past Epstein‐Barr virus infection in risk of initial CNS demyelination. Neurology 2011;77:371–379. 21753179 10.1212/WNL.0b013e318227062a

[30] van der Mei IA , Ponsonby AL , Dwyer T , et al. Vitamin D levels in people with multiple sclerosis and community controls in Tasmania, Australia. J Neurol 2007;254:581–590. 17426912 10.1007/s00415-006-0315-8

[31] Hedström AK , Huang J , Michel A , et al. High levels of Epstein‐Barr virus nuclear Antigen‐1‐specific antibodies and infectious mononucleosis act both independently and synergistically to increase multiple sclerosis risk. Front Neurol 2019;10:1368. 32038456 10.3389/fneur.2019.01368 PMC6992610

[32] Brenner N , Mentzer AJ , Butt J , et al. Validation of multiplex serology detecting human herpesviruses 1‐5. PLoS One 2018;13:e0209379. 30589867 10.1371/journal.pone.0209379 PMC6307738

[33] Hedstrom AK , Olsson T , Kockum I , et al. Low sun exposure increases multiple sclerosis risk both directly and indirectly. J Neurol 2020;267:1045–1052. 31844981 10.1007/s00415-019-09677-3 PMC7109160

[34] Dilthey A , Leslie S , Moutsianas L , et al. Multi‐population classical HLA type imputation. PLoS Comput Biol 2013;9:e1002877. 23459081 10.1371/journal.pcbi.1002877 PMC3572961

[35] VanderWeele TJ , Knol MJ . A tutorial on interaction. Epidemiol Methods 2014;3:33–72.

[36] VanderWeele TJ . On the distinction between interaction and effect modification. Epidemiology 2009;20:863–871. 19806059 10.1097/EDE.0b013e3181ba333c

[37] Galea S , Riddle M , Kaplan GA . Causal thinking and complex system approaches in epidemiology. Int J Epidemiol 2010;39:97–106. 19820105 10.1093/ije/dyp296 PMC2912489

[38] Valeri L , Vanderweele TJ . Mediation analysis allowing for exposure‐mediator interactions and causal interpretation: theoretical assumptions and implementation with SAS and SPSS macros. Psychol Methods 2013;18:137–150. 23379553 10.1037/a0031034 PMC3659198

[39] Maksimovic J , Phipson B , Oshlack A . A cross‐package Bioconductor workflow for analysing methylation array data. F1000Res 2016;5:1281. 27347385 10.12688/f1000research.8839.1 PMC4916993

[40] Teschendorff AE , Breeze CE , Zheng SC , Beck S . A comparison of reference‐based algorithms for correcting cell‐type heterogeneity in epigenome‐wide association studies. BMC Bioinformatics 2017;18:105. 28193155 10.1186/s12859-017-1511-5 PMC5307731

[41] Benjamini Y , Hochberg Y . Controlling the false discovery rate: a practical and powerful approach to multiple testing. J R Stat Soc Series B Stat Methodol 1995;57:289–300.

[42] Zhang B , Horvath S . A general framework for weighted gene co‐expression network analysis. Stat Appl Genet Mol Biol 2005;4:Article17. 16646834 10.2202/1544-6115.1128

[43] Ruiz‐Perez D , Guan H , Madhivanan P , et al. So you think you can PLS‐DA? BMC bioinformatics 2020;21:2. 33297937 10.1186/s12859-019-3310-7 PMC7724830

[44] Graeber TG . Hypergeometric Test Calculator, Available at: https://systems.crump.ucla.edu/hypergeometric/index.php. Los Angeles, USA2009.

[45] Qin Q , Fan J , Zheng R , et al. Lisa: inferring transcriptional regulators through integrative modeling of public chromatin accessibility and ChIP‐seq data. Genome Biol 2020;21:1–14. 10.1186/s13059-020-1934-6 PMC7007693 32033573

[46] Ochoa D , Hercules A , Carmona M , et al. The next‐generation open targets platform: reimagined, redesigned, rebuilt. Nucleic Acids Res 2023;51:D1353–D1359. 36399499 10.1093/nar/gkac1046 PMC9825572

[47] von Elm E , Altman DG , Egger M , et al. The strengthening the reporting of observational studies in epidemiology (STROBE) statement: guidelines for reporting observational studies. Lancet (London, England) 2007;370:1453–1457. 18064739 10.1016/S0140-6736(07)61602-X

[48] van der Mei I , Lucas RM , Taylor BV , et al. Population attributable fractions and joint effects of key risk factors for multiple sclerosis. Mult Scler 2016;22:461–469. 26199349 10.1177/1352458515594040

[49] Bjornevik K , Cortese M , Healy BC , et al. Longitudinal analysis reveals high prevalence of Epstein‐Barr virus associated with multiple sclerosis. Science 2022;375:296–301. 35025605 10.1126/science.abj8222

[50] Hedstrom AK . Risk factors for multiple sclerosis in the context of Epstein‐Barr virus infection. Front Immunol 2023;14:1212676. 37554326 10.3389/fimmu.2023.1212676 PMC10406387

[51] Dwyer T , Couper D , Walter SD . Sources of heterogeneity in the meta‐analysis of observational studies: the example of SIDS and sleeping position. J Clin Epidemiol 2001;54:440–447. 11337206 10.1016/s0895-4356(00)00313-9

[52] Drosu N , Anderson M , Bilodeau PA , et al. CD4 T cells restricted to DRB1*15:01 recognize two Epstein‐Barr virus glycoproteins capable of intracellular antigen presentation. Proc Natl Acad Sci U S A 2024;121:e2416097121. 39432795 10.1073/pnas.2416097121 PMC11536159

[53] Bosco EE , Mulloy JC , Zheng Y . Rac1 GTPase: a “Rac” of all trades. Cell Mol Life Sci 2009;66:370–374. 19151919 10.1007/s00018-008-8552-x PMC6669905

[54] Christopoulos PF , Gjolberg TT , Kruger S , et al. Targeting the Notch signaling pathway in chronic inflammatory diseases. Front Immunol 2021;12:668207. 33912195 10.3389/fimmu.2021.668207 PMC8071949

[55] Anderson LJ , Longnecker R . An auto‐regulatory loop for EBV LMP2A involves activation of Notch. Virology 2008;371:257–266. 17980397 10.1016/j.virol.2007.10.009 PMC2288488

[56] Chen Y , Fachko D , Ivanov NS , et al. Epstein‐Barr virus microRNAs regulate B cell receptor signal transduction and lytic reactivation. PLoS Pathog 2019;15:e1007535. 30615681 10.1371/journal.ppat.1007535 PMC6336353

[57] Raftopoulou S , Rapti A , Karathanasis D , et al. The role of type I IFN in autoimmune and autoinflammatory diseases with CNS involvement. Front Neurol 2022;13:1026449. 36438941 10.3389/fneur.2022.1026449 PMC9685560

[58] Bellucci G , Albanese A , Rizzi C , et al. The value of interferon β in multiple sclerosis and novel opportunities for its anti‐viral activity: a narrative literature review. Front Immunol 2023;14:1161849. 37334371 10.3389/fimmu.2023.1161849 PMC10275407

[59] Stewart N , Simpson SL Jr , van der Mei I , et al. Interferon‐beta and serum 25‐hydroxyvitamin D interact to modulate relapse risk in MS. Neurology 2012;79:254–260. 22700816 10.1212/WNL.0b013e31825fded9

[60] Hüls A , Ickstadt K , Schikowski T , Krämer U . Detection of gene‐environment interactions in the presence of linkage disequilibrium and noise by using genetic risk scores with internal weights from elastic net regression. BMC Genet 2017;18:1–14. 28606108 10.1186/s12863-017-0519-1 PMC5469185

[61] Zhang H , Zheng Y , Zhang Z , et al. Estimating and testing high‐dimensional mediation effects in epigenetic studies. Bioinformatics 2016;32:3150–3154. 27357171 10.1093/bioinformatics/btw351 PMC5048064

[62] Vanderweele TJ , Vansteelandt S . Odds ratios for mediation analysis for a dichotomous outcome. Am J Epidemiol 2010;172:1339–1348. 21036955 10.1093/aje/kwq332 PMC2998205

[63] Rijnhart JJM , Lamp SJ , Valente MJ , et al. Mediation analysis methods used in observational research: a scoping review and recommendations. BMC Med Res Methodol 2021;21:226. 34689754 10.1186/s12874-021-01426-3 PMC8543973

[64] Gustafsson M , Nestor CE , Zhang H , et al. Modules, networks and systems medicine for understanding disease and aiding diagnosis. Genome Med 2014;6(10):82. 25473422 10.1186/s13073-014-0082-6 PMC4254417

[65] Yu X , Zhao H , Wang R , et al. Cancer epigenetics: from laboratory studies and clinical trials to precision medicine. Cell Death Discov 2024;10:28. 38225241 10.1038/s41420-024-01803-z PMC10789753

[66] Borde C , Quignon F , Amiel C , et al. Methyl‐qPCR: a new method to investigate Epstein‐Barr virus infection in post‐transplant lymphoproliferative diseases. Clin Epigenetics 2022;14:33. 35246247 10.1186/s13148-022-01255-1 PMC8895795

