# Supplementary material for: Epstein–Barr Virus, Lower Vitamin D, Low Sun Exposure, and HLA‐DRB1*1501 Risk Variant Share Common Epigenetic Pathways Leading to Multiple Sclerosis Onset
Source: Ann Neurol. 2025 Oct 10;99(2):341–55. doi: 10.1002/ana.78043 (PMC12894492; doi:10.1002/ana.78043)
Supplement: Supplementary file 1 — Supplementary Data S1 Supporting Information. [file ANA-99-341-s002.docx]

Supporting Information for

Epstein-Barr virus, lower vitamin D, low sun exposure, and *HLA-DRB1*1501* share common epigenetic pathways leading to multiple sclerosis onset

Steve Simpson-Yap^1,2,3,†^ & Ellen Morwitch^1,†^, Samuel A Tanner^1,4,5^, Sarah M Thomson^1^, Alex Eisner^1^, Rod A Lea^6,7,8^, Trevor J Kilpatrick^1^, Jeannette Lechner-Scott^7,8,9^, Rodney J Scott^8,10^, Alexandre Xavier^8,10^, Vicki E Maltby^7,8,9^, Robyn M Lucas^11^, Bruce V Taylor^3^, Brett A Lidbury^11^, Simon A Broadley^12^, Ingrid van der Mei^3^, Mehari Woldemariam Merid^1,13^, Boris Novakovic^4,14^, Richard Saffery^4,14^, Anna Karin Hedström^15^, Pernilla Stridh^15^, Tomas Olsson^15^, Maja Jagodic^15,16,17^, Lars Alfredsson^15,16,17^, Ausimmune Investigator Group^18^, and Anne-Louise Ponsonby^1,4^

^†^These authors contributed equally to this work.

Author affiliations:

1. The Florey Institute of Neuroscience and Mental Health, The University of Melbourne, Parkville, Australia;
2. Neuroepidemiology Unit, Melbourne School of Population & Global Health, The University of Melbourne, Parkville, Australia;
3. MS Research Flagship, Menzies Institute for Medical Research, University of Tasmania, Hobart, Australia;
4. Murdoch Children’s Research Institute, The University of Melbourne, Parkville, Australia;
5. Melbourne Bioinformatics, The University of Melbourne, Parkville, VIC, Australia;
6. Centre for Genomics and Personalised Health, School of Biomedical Science, Queensland University of Technology, Kelvin Grove, Australia;
7. School of Medicine and Public Health, The University of Newcastle, Callaghan, Australia;
8. Hunter Medical Research Institute, The University of Newcastle, Callaghan, Australia;
9. Department of Neurology, John Hunter Hospital, New Lambton Heights, Australia;
10. School of Biomedical Sciences and Pharmacy, The University of Newcastle, Callaghan, Australia;
11. National Centre for Epidemiology and Population Health, The Australian National University, Acton, Australia;
12. School of Medicine and Dentistry, Gold Coast Campus, Griffith University, Southport, Australia;
13. Department of Paediatrics, The Royal Children’s Hospital, Parkville, Australia;
14. Department of Clinical Neuroscience, Karolinska Institutet, Stockholm, Sweden;
15. Center for Molecular Medicine, Karolinska University Hospital, Stockholm, Sweden;
16. Institute of Environmental Medicine, Karolinska Institutet, Stockholm, Sweden;
17. The full list of people in the Ausimmune Investigators Group is included in the Acknowledgements.

Contents:

[Supplemental Figure S1. Ausimmune: WGCNA DNAm module dendrogram 3](#_Toc205896346)

[Supplemental Figure S2. Ausimmune: scale independence and mean connectivity pre-WGCNA. 3](#_Toc205896347)

[Supplemental Figure S3. The association between higher anti-EBNA IgG levels and MS risk is potentiated by lower serum 25(OH)D levels. 4](#_Toc205896348)

[Supplemental Table S1. Ausimmune analysis sample characteristics. 5](#_Toc205896349)

[Supplemental Table S2. EIMS analysis sample characteristics. 8](#_Toc205896350)

[Supplemental Table S3. Ausimmune: EBV and sun/vitamin D positively interact in association with MS onset. 11](#_Toc205896351)

[Supplemental Table S4. Associations between DNAm modules and MS in Ausimmune and EIMS. 16](#_Toc205896352)

[Supplemental Table S5. Ausimmune and EIMS: Associations between environmental risk factors and DNAm modules in each study. 18](#_Toc205896353)

[Supplemental Table S6. Sensitivity Analysis - Ausimmune: Mediation of selected environmental/lifestyle factors by A1-module and A2-module, restricted to FDEs. 22](#_Toc205896354)

[Supplemental Table S7. Sensitivity analysis - Ausimmune: Mediation of selected environmental/lifestyle factors by A1-module and A2-module DNAm modules, restricted to cases not being treated with disease-modifying therapies (DMTs) at time of survey. 25](#_Toc205896355)

[Supplemental Table S8. EIMS: Mediation of exposure-MS associations by DNAm modules. 27](#_Toc205896356)

[Supplemental Table S9. Sensitivity analysis - EIMS: Mediation of exposure-MS associations by DNAm modules, restricted to cases not being treated with disease-modifying therapies (DMTs) at time of survey. 30](#_Toc205896357)

[Supplemental Table S10. Sensitivity analysis - EIMS: Mediation of exposure-MS associations by DNAm modules, restricted to cases up to 5 years post-MS onset at time of survey. 33](#_Toc205896358)

[Supplemental Table S11. Sensitivity analysis, Ausimmune- reverse (MS-to-risk factor) mediation 36](#_Toc205896359)

[Supplemental Table S12. Sensitivity analysis, EIMS-reverse (MS-to-risk factor) mediation 37](#_Toc205896360)

[Supplemental Table S13. pathfindR enrichment results for pathways common to Ausimmune and EIMS DNAm modules: Reactome. 40](#_Toc205896361)

[Supplemental Table S14. pathfindR enrichment results for pathways common to Ausimmune and EIMS DNAm modules: Gene Ontology. 48](#_Toc205896362)

**Supplemental Figure S1**. Ausimmune: WGCNA DNAm module dendrogram


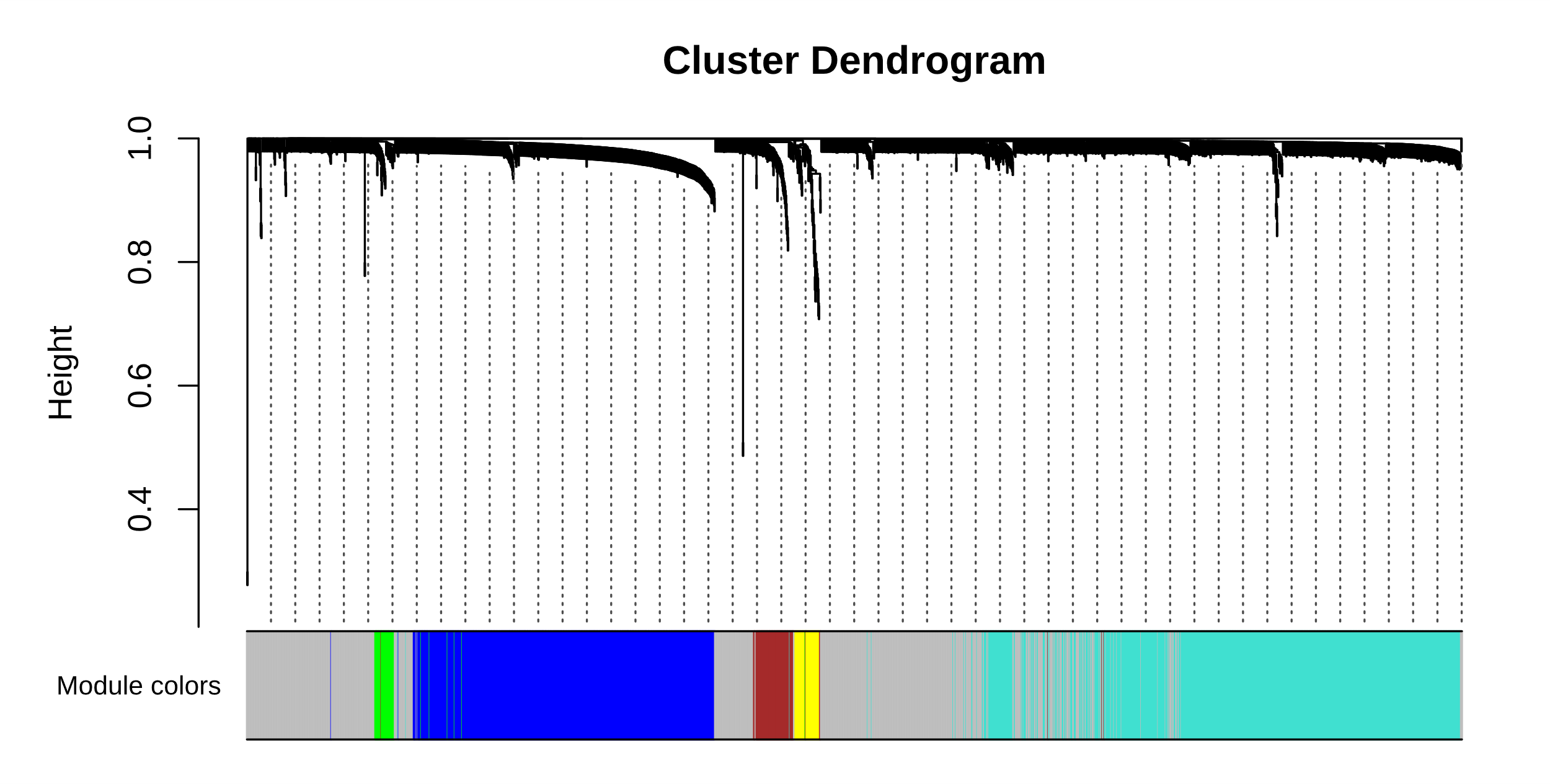


**Supplemental Figure S2.** Ausimmune: scale independence and mean connectivity pre-WGCNA.


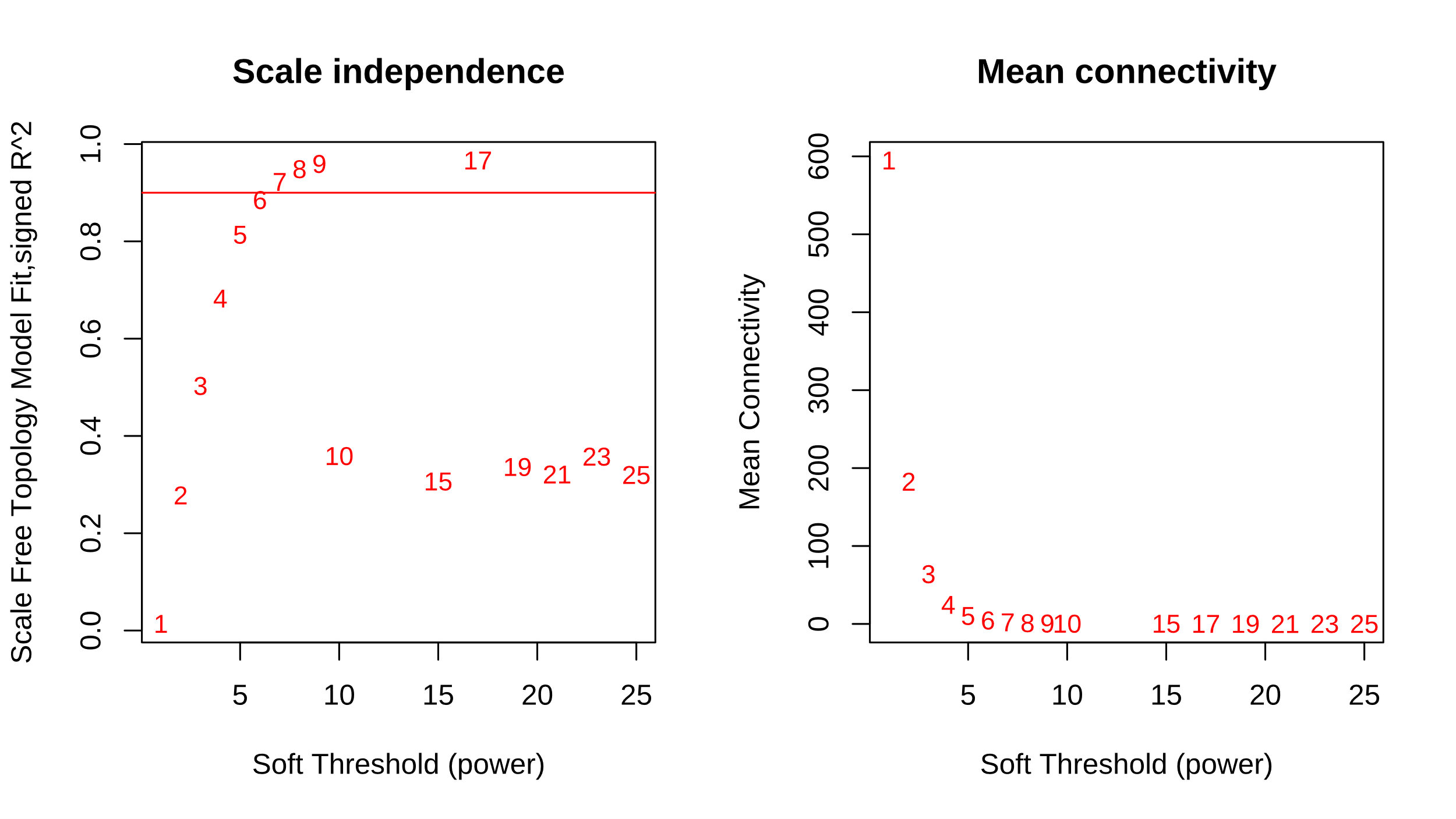


**Supplemental Figure S3.** The association between higher anti-EBNA IgG levels and MS risk is potentiated by lower serum 25(OH)D levels.


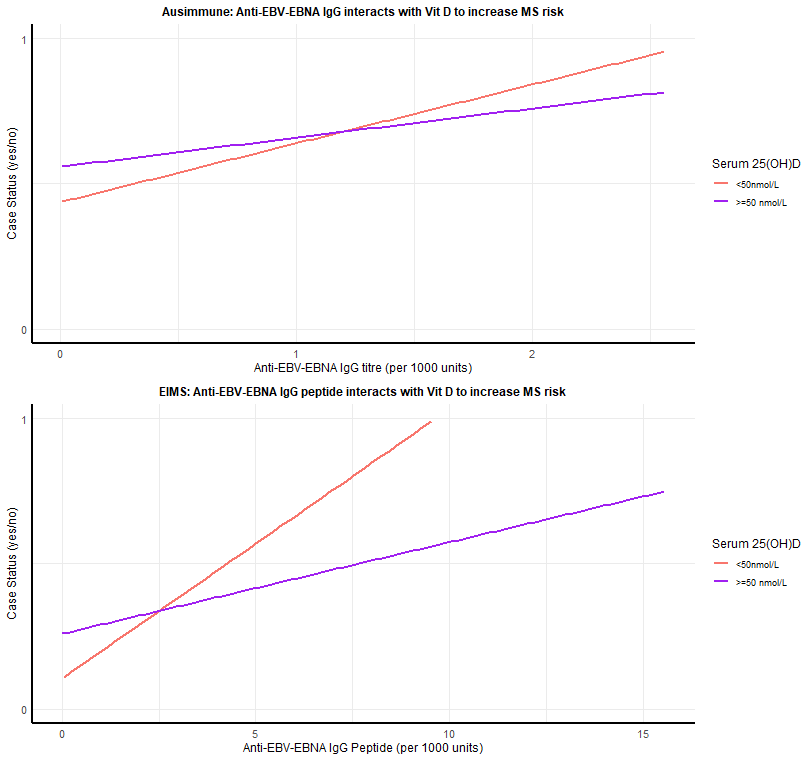


Note: for each study there is a stronger association between increasing anti-EBNA IgG titer and MS risk among those with low 25(OH)D (<50 nmol/L) compared to those with higher 25(OH)D ($\boldsymbol{\geq}$50 nmol/L). Interaction Odds Ratios for a per 1000 unit increase in the EBNA measure, and 25(OH)D<50 nmol/L vs ≥50 nmol/L Ausimmune: OR=1.95 (1.17, 3.62) p=0.022. EIMS: OR=1.21 (1.14, 1.30) p=1.44x10-8.

**Supplemental Table S1.** Ausimmune analysis sample characteristics.

|  | [n (%)] or [median (interquartile range)] | |  |  |
| --- | --- | --- | --- | --- |
|  | Controls (n=348) | Cases (n=206) | OR (95% CI) | P value |
| FCD onset type |  |  |  |  |
| FDE |  | 107 (51.9%) |  |  |
| FCD with historical |  | 84 (40.8%) |  |  |
| Progressive-onset |  | 15 (7.3%) |  |  |
| Disease-modifying therapy use |  |  |  |  |
| No |  | 163 (79.1%) |  |  |
| Yes |  | 43 (20.9%) |  |  |
| Age, years | 39.71 (32.51-47.62) | 38.80 (31.35-46.55) | 0.99 (0.97, 1.00) | 0.13 |
| Sex |  |  |  |  |
| Male | 75 (21.3%) | 45 (21.8%) | 1.00 [Reference] |  |
| Female | 273 (78.7%) | 161 (78.2%) | 0.97 (0.64, 1.47) | 0.89 |
| Study region |  |  |  |  |
| Brisbane City, QLD | 139 (40.1%) | 75 (36.4%) | 1.00 [Reference] |  |
| Newcastle city & surrounds, NSW | 35 (10.1%) | 21 (10.2%) | 1.11 (0.60, 2.05) | 0.73 |
| Geelong city and the Western Districts, VIC | 100 (28.8%) | 53 (25.7%) | 0.98 (0.64, 1.52) | 0.94 |
| State of Tasmania | 73 (21.0%) | 57 (27.7%) | 1.45 (0.93, 2.26) | 0.10 |
| Ever smoked? |  |  |  |  |
| No | 168 (48.7%) | 74 (36.3%) | 1.00 [Reference] |  |
| Yes | 177 (51.3%) | 130 (63.7%) | 1.72 (1.20, 2.46) | 0.003 |
| History of infectious mononucleosis |  |  |  |  |
| No | 292 (84.6%) | 142 (69.6%) | 1.00 [Reference] |  |
| Yes | 53 (15.4%) | 62 (30.4%) | 2.36 (1.54, 3.60) | 0.001 |
| Anti-EBNA IgG titer (dichotomized) |  |  |  |  |
| <640 | 127 (77.9%) | 108 (64.7%) | 1.00 [Reference] |  |
| $\boldsymbol{\geq}$640 (top quartile) | 36 (22.1%) | 59 (35.3%) | 2.11 (1.22, 3.65) | 0.008 |
| Deseasonalized serum 25(OH)D (nmol/L) | 79.16 (62.96-99.28) | 75.42 (55.51-93.42) | 0.92 (0.86, 0.98) | 0.010 |
| Summer sun exposure in the 3 years before onset |  |  |  |  |
| ≥2 hours/day | 181 (52.6%) | 85 (41.5%) | 1.00 [Reference] |  |
| <2 hours/day | 163 (47.4 %) | 120 (58.5%) | 1.74 (1.20, 2.50) | 0.003 |
| *HLA-DRB1*1501* variant |  |  |  |  |
| GG | 248 (71.7%) | 91 (44.2%) | 1.00 [Reference] |  |
| AA/AG | 98 (28.3%) | 115 (55.8%) | 3.33 (2.31, 4.83) | <0.001 |
| Abbreviations: 25(OH)D=25-hydroxyvitamin D; EBNA=Epstein-Barr Nuclear Antigen; FCD=first clinical demyelination; FDE=first clinical demyelinating event; HLA=human leucocyte antigen; nmol/L=nanomole/liter; NSW=New South Wales; QLD=Queensland; VIC=Victoria. HLA-DRB1*1501 risk genotype (rs3135388) is AA/AG vs GG (risk).  Tests for difference by logistic regression. Results in boldface denote statistical significance (p<0.05).  Note – because only 347 of the 348 Ausimmune controls had exposure data available, only these are included in exposure-outcome, exposure-mediator, and mediation analyses. However, Table S1 and the overall sample characteristics refer to the 348 controls for whom DNAm modules were estimated, and this additional person also contributes to the estimations of the DNAm module scores. | | | | |

**Supplemental Table S2**. EIMS analysis sample characteristics.

|  | [n (%)] or [median (interquartile range)] | |  |  |
| --- | --- | --- | --- | --- |
|  | Controls (n=139) | Cases (n=140) | aOR (95%CI)^a^ | P value |
| Disease-modifying therapy use |  |  |  |  |
| No |  | 52 (37.1%) |  |  |
| Yes |  | 88 (62.9%) |  |  |
| Age, years | 35.6 (10.0; 14-62) | 33.6 (10.2; 15-61) | 0.98 (0.95, 1.00) | 0.062 |
| Sex |  |  |  |  |
| Male | 35 (25.2%) | 42 (30.0%) | 1.00 [Reference] |  |
| Female | 104 (74.8%) | 98 (70.0%) | 0.77 (0.45, 1.31) | 0.33 |
| Smoking status |  |  |  |  |
| Never | 72 (51.8%) | 69 (49.3%) | 1.00 [Reference] |  |
| Ex | 39 (28.1%) | 47 (33.6%) | 1.46 (0.84, 2.56) |  |
| Current | 28 (20.1%) | 24 (17.1%) | 0.85 (0.45, 1.63) | 0.63 |
| History of Infectious mononucleosis |  |  |  |  |
| No | 124 (91.2%) | 119 (85.0%) | 1.00 [Reference] |  |
| Yes | 15 (10.8%) | 21 (15.0%) | 1.52 (0.74, 3.15) | 0.39 |
| Anti-EBNA peptide segment (AA 385-420) IgG |  |  |  |  |
| Continuous (mean (SD; range)) | 5,423.6 (3,393.8; 1-15,563) | 7,331.9 (2,931.1; 262.1-14,373) | 1.02 (1.01, 1.03) | <0.001 |
| Dichotomized at median (6716) |  |  |  |  |
| Lower | 83 (64.3%) | 50 (36.8%) | 1.00 [Reference] |  |
| Higher | 46 (35.7%) | 86 (63.2%) | 3.00 (1.80, 4.99) | <0.001 |
| Deseasonalized serum 25(OH)D (nmol/L) | 73.9 (24.9; 13-138) | 64.3 (28.5; 10-136) | 0.98 (0.98, 0.99) | 0.001 |
| Low sun-seeking behaviours^b^ |  |  |  |  |
| 3-6 | 72 (51.8%) | 72 (51.85) | 1.00 [Reference] |  |
| 7-12 | 67 (48.2%) | 67 (48.2%) | 0.94 (0.58, 1.52) | 0.80 |
| *HLA-DRB1*1501* |  |  |  |  |
| GG | 97 (69.8%) | 55 (39.3%) | 1.00 [Reference] |  |
| AG/AA | 42 (30.2%) | 85 (60.7%) | 3.79 (2.30, 6.34) | <0.001 |
| Abbreviations: 25(OH)D=25-hydroxyvitamin D; EBNA=EBV-Epstein Barr Nuclear Antigen; HLA=Human Leucocyte Antigen; nmol/L=nanomole/liter; SD=standard deviation; Sun combined=sunbathing, travel to sunnier countries and sunbed use. Tests for difference by logistic regression. a. Model adjusted for age, sex, and smoking status. b. Low sun exposure in EIMS is defined from responses to three self-reported questions regarding sun exposure (frequency of sunbathing, frequency of recent travel to a sunnier country than Sweden, and frequency of use of sunbeds), estimating a composite score ranging 3-12, here dichotomized and low sun exposure defined as sun score≤6. *HLA-DRB1*1501* risk genotype (rs3135388) is AA/AG vs GG (risk).  Tests for difference by logistic regression. Results in boldface denote statistical significance (p<0.05). | | | | |

**Supplemental Table S3**. Ausimmune: EBV and sun/vitamin D positively interact in association with MS onset.

|  | Cases/controls  (% cases) | aOR (95%CI) | RERI (95%CI) | AP (95%CI) |
| --- | --- | --- | --- | --- |
| Anti-EBNA IgG - Summer sun |  |  |  |  |
| >2-3hr/day, EBNA^non-risk^ | 15/37 (28.9%) | 1.00 [Reference] |  |  |
| ≤2-3hr/day, EBNA^non-risk^ | 22/30 (42.3%) | 1.96 (0.85, 4.52) |  |  |
| >2-3hr/day, EBNA^risk^ | 52/44 (54.2%) | 3.05 (1.47, 6.37) |  |  |
| ≤2-3hr/day, EBNA^risk^ | 78/49 (61.4%) | 4.48 (2.16, 9.32) | 0.47 (-1.89, 2.84) | 0.11 (-0.41, 0.62) |
| Trend: |  | *p<0.001* | *p=0.70* | *p=0.69* |
| Infectious mononucleosis (IM)- Summer sun |  |  |  |  |
| >2-3hr/day, IM^no^ | 54/157 (74.4%) | 1.00 [Reference] |  |  |
| ≤2-3hr/day, IM^no^ | 87/133 (60.5%) | 2.13 (1.39, 3.26) |  |  |
| >2-3hr/day, IM^yes^ | 28/23 (54.9%) | 3.64 (1.93, 6.87) |  |  |
| ≤2-3hr/day, IM^yes^ | 33/28 (54.1%) | 3.60 (1.97, 6.58) | -1.17 (-4.03, 1.69) | -0.32 (-1.22, 0.57) |
| Trend: |  | *p<0.001* | *p=0.42* | *p=0.48* |
| Anti-EBNA IgG - Winter sun |  |  |  |  |
| >2-3hr/day, EBNA^non-risk^ | 12/33 (26.7%) | 1.00 [Reference] |  |  |
| ≤2-3hr/day, EBNA^non-risk^ | 25/34 (42.4%) | 2.27 (0.96, 5.37) |  |  |
| >2-3hr/day, EBNA^risk^ | 45/41 (52.3%) | 3.23 (1.46, 7.16) |  |  |
| ≤2-3hr/day, EBNA^risk^ | 85/52 (62.0%) | 5.36 (2.45, 11.73) | 0.86 (-1.81, 3.52) | 0.16 (-0.31, 0.63) |
| Trend: |  | *p<0.001* | *p=0.53* | *p=0.51* |
| Infectious mononucleosis (IM)- Winter sun |  |  |  |  |
| >2-3hr/day, IM^no^ | 46/140 (24.7%) | 1.00 [Reference] |  |  |
| ≤2-3hr/day, IM^no^ | 95/150 (38.8%) | 2.07 (1.34, 3.21) |  |  |
| >2-3hr/day, IM^yes^ | 23/19 (54.8%) | 3.74 (1.86, 7.55) |  |  |
| ≤2-3hr/day, IM^yes^ | 38/32 (54.3%) | 3.69 (2.06, 6.62) | -1.12 (-4.14, 1.90) | -0.30 (-1.20, 0.59) |
| Trend: |  | *p<0.001* | *p=0.47* | *p=0.51* |
| Anti-EBNA IgG - 25(OH)D |  |  |  |  |
| >50nmol/L, EBNA^non-risk^ | 32/60 (34.5%) | 1.00 [Reference] |  |  |
| ≤50nmol/L EBNA^non-risk^ | 5/8 (38.5%) | 1.24 (0.37, 4.16) |  |  |
| >50nmol/L, EBNA^risk^ | 110/85 (56.4%) | 2.54 (1.50, 4.28) |  |  |
| ≤50nmol/L, EBNA^risk^ | 20/9 (69.0%) | 4.48 (1.78, 11.25) | 1.70 (-2.21, 5.62) | 0.38 (-0.23, 0.99) |
| Trend: |  | *p<0.001* | *p=0.39* | *p=0.22* |
| Infectious mononucleosis (IM)- 25(OH)D |  |  |  |  |
| >50nmol/L, IM^no^ | 114/262 (30.3%) | 1.00 [Reference] |  |  |
| ≤50nmol/L, IM^no^ | 28/29 (49.1%) | 2.19 (1.23, 3.89) |  |  |
| >50nmol/L, IM^yes^ | 54/46 (54.0%) | 2.67 (1.69, 4.21) |  |  |
| ≤50nmol/L, IM^yes^ | 7/5 (58.3%) | 2.89 (0.88, 9.49) | -0.96 (-4.69, 2.77) | -0.33 (-1.97, 1.31) |
| Trend: |  | *p<0.001* | *p=0.61* | *p=0.69* |
| Anti-EBNA IgG - 25(OH)D |  |  |  |  |
| 80nmol/L, EBNA^non-risk^ | 19/35 (35.2%) | 1.00 [Reference] |  |  |
| ≤80nmol/L, EBNA^non-risk^ | 18/33 (35.3%) | 1.04 (0.45, 2.37) |  |  |
| >80nmol/L, EBNA^risk^ | 60/50 (54.6%) | 2.30 (1.17, 4.55) |  |  |
| ≤80nmol/L, EBNA^risk^ | 70/44 (61.4%) | 3.14 (1.55, 6.34) | 0.80 (-0.83, 2.42) | 0.25 (-0.22, 0.73) |
| Trend: |  | *p<0.001* | *p=0.34* | *p=0.30* |
| Infectious mononucleosis (IM)- 25(OH)D |  |  |  |  |
| >80nmol/L, IM^no^ | 61/140 (30.4%) | 1.00 [Reference] |  |  |
| ≤80nmol/L, IM^no^ | 81/151 (34.9%) | 1.20 (0.78, 1.84) |  |  |
| >80nmol/L, IM^yes^ | 29/25 (53.7%) | 2.65 (1.43, 4.92) |  |  |
| ≤80nmol/L, IM^yes^ | 32/26 (55.2%) | 2.68 (1.46, 4.94) | -0.16 (-2.22, 1.90) | -0.06 (-0.85, 0.73) |
| Trend: |  | *p<0.001* | *p=0.88* | *p=0.88* |
| Abbreviations: aOR=adjusted odds ratio; 95%CI=95% confidence interval; EBNA=Epstein Barr Nuclear Antigen; IM=Infectious Mononucleosis; 25(OH)D=25-hydroxyvitamin D; RERI=Relative Excess Risk due to Interaction; Analyses by logistic regression, estimating aOR (95% CI). 25(OH)D is seasonally adjusted. Results in boldface denote statistical significance (p<0.05). Additional analyses stratified by *HLA-DR15* genotype found no differences (data not shown). EBNA^risk^$\boldsymbol{\geq}$160; EBNA^non-risk^<160; IM^no^= have not had IM or not sure; IM^yes^= have had IM. | | | | |

**Supplemental Table S4**. Associations between DNAm modules and MS in Ausimmune and EIMS.

|  | Full sample | | DMT treatment naïve | | Restricted to FDEs (Ausimmune-only) | |
| --- | --- | --- | --- | --- | --- | --- |
|  | aOR (95%CI) | p-value | aOR (95%CI) | p-value | aOR (95%CI) | p-value |
| Ausimmune |  |  |  |  |  |  |
| *A1-module* | 6.00x10^14^ (9.37x10^11^, 6.57x10^17^) | 3.30x10^-23^ | 9.68×10^13^ (1.11×10^11^, 1.52×10^17^) | 3.7×10^-19^ | 9.76x10^13^ (4.11x10^10^, 4.92x10^17^) | 8.27x10^-15^ |
| *A2-module* | 1.68x10^12^ (4.79x10^9^, 9.3x10^14^) | 1.13x10^-19^ | 1.25×10^12^ (2.15×10^9^, 1.21×10^15^) | 1.47×10^-16^ | 1.20x10^11^ (8.90x10^7^, 3.12x10^14^) | 2.89x10^-11^ |
| *A3-module* | 2.85x10^7^ (2.55x10^5^, 4.10x10^9^) | 3.45x10^-12^ | 1.18×10^7^ (6.92×10^4^, 2.62×10^9^) | 1.32×10^-9^ | 1.49 × 10^6^ (4.13×10^3^, 7.27×10^8^) | 3.8×10^-6^ |
| *A4-module* | 6.50x10^6^ (6.02x10^4^, 8.77x10^8^) | 1.32x10^-10^ | 2.24×10^6^ (1.63×10^4^, 3.88×10^8^) | 1.22×10^-8^ | 6.56×10^6^ (2.08×10^4^, 2.74 × 10^9^) | 1.68×10^-7^ |
| *A5-module* | 1.48x10^6^ (1.67x10^4^, 1.59x10^8^) | 1.13x10^-9^ | 1.34×10^7^ (8.1×10^4^, 2.85×10^9^) | 7.56×10^-10^ | 8.33×10^5^ (2.65×10^3^, 3.38×10^8^) | 5.21×10^-6^ |
| EIMS |  |  |  |  |  |  |
| *E1-module* | 5.71 (3.54, 9.67) | 1.03x10^-11^ | 5.51 (2.87, 11.50) | 1.25x10^-6^ |  |  |
| *E2-module* | 8.99 (4.16, 20.4) | 5.50x10^-8^ | 7.46 (2.61, 22.90) | 2.67x10^-4^ |  |  |
| *E3-module* | 1.72 (0.38, 8.10) | 0.49 | 4.63 (0.49, 46.30) | 0.18 |  |  |
| Abbreviations: aOR=adjusted odds ratio; 95%CI=95% confidence interval; DMT=disease-modifying therapy; FDE=first demyelinating event. Ausimmune models adjusted for age, sex, and study region. EIMS models adjusted for age, sex, and smoking status. Results in boldface denote statistical significance (p<0.05). | | | | | | |

**Supplemental Table S5.** Ausimmune and EIMS: Associations between environmental risk factors and DNAm modules in each study.

| Ausimmune | A1-module | | A2-module | |  |  |
| --- | --- | --- | --- | --- | --- | --- |
|  | β (95%CI) | p-value | β (95%CI) | p-value |  |  |
| History of Infectious Mononucleosis (yes vs no) | 0.0119  (0.0031, 0.0207) | 0.008 | 0.0058  (-0.0032, 0.0147) | 0.21 |  |  |
| Anti-EBNA IgG titer (top quartile vs rest)^a^ | 0.0114  (0.0014, 0.0213) | 0.026 | 0.0079  (-0.0024, 0.0182) | 0.14 |  |  |
| Lower 25(OH)D^b^ | 0.0014  (0.0002, 0.0027) | 0.026 | 0.0012  (0.001, 0.0026) | 0.033 |  |  |
| Low summer sun^c^ | 0.0109  (0.0036, 0.0182) | 0.004 | 0.0115  (0.0043, 0.0180) | 0.002 |  |  |
| *HLA-DRB1*1501* (rs3135388, AA/AG vs GG)^d^ | 0.0146  (0.00739, 0.0218) | <0.001 | 0.0113  (0.0041, 0.0186) | 0.002 |  |  |
|  | A3-module | | A4-module | | A5-module | |
|  | β (95%CI) | p-value | β (95%CI) | p-value | β (95%CI) | p-value |
| History of Infectious Mononucleosis (yes vs no) | 0.0009  (-0.0080, 0.0097) | 0.85 | 0.0065  (-0.0021, 0.0150) | 0.14 | 0.0068  (-0.0021, 0.0160) | 0.13 |
| EBNA IgG titer (top quartile vs rest)^a^ | 0.0067  (-0.0038, 0.0170) | 0.21 | 0.0055  (-0.0041, 0.0150) | 0.26 | 0.0049  (-0.0058, 0.0160) | 0.37 |
| Lower 25(OH)D^b^ | 0.0011  (-0.0001, 0.0024) | 0.082 | 0.0006  (-0.0006, 0.0019) | 0.30 | 0.0009  (-0.0004, 0.0022) | 0.16 |
| Low summer sun^c^ | 0.0100  (0.0028, 0.0170) | 0.007 | 0.0037  (-0.0035, 0.0110) | 0.31 | 0.0072  (-0.0001, 0.0140) | 0.055 |
| *HLA-DRB1*1501* (rs3135388, AA/AG vs GG)^d^ | 0.0100  (0.0031, 0.0180) | 0.005 | 0.0081  (0.0010, 0.0150) | 0.025 | 0.0067  (-0.0007, 0.0140) | 0.076 |
|  | E1-module | | E2-module | |  |  |
| EIMS | β (95%CI) | p-value | β (95%CI) | p-value |  |  |
| History of Infectious Mononucleosis (yes vs no) | 0.018  (-0.23, 0.26) | 0.89 | 0.130  (0.007, 0.250) | 0.039 |  |  |
| Anti-EBNA IgG peptide (above median vs rest)^e^ | 0.320  (0.16, 0.49) | <0.001 | 0.082  (-0.003, 0.170) | 0.059 |  |  |
| Lower 25(OH)D^b^ | 0.028  (-0.005, 0.061) | 0.093 | 0.018  (0.001, 0.034) | 0.036 |  |  |
| Low sun-seeking behaviours^f^ | 0.130  (-0.035, 0.29) | 0.13 | -0.068  (-0.15, 0.014) | 0.11 |  |  |
| *HLA-DRB1*1501* (rs3135388, AA/AG vs GG)^d^ | 0.74  (0.52, 0.96) | <0.001 | 1.30  (1.16, 1.51) | <0.001 |  |  |
| Abbreviations: EBNA=Epstein Barr Nuclear Antigen; 25(OH)D=25-hydroxyvitamin D; HLA=Human Leucocyte Antigen.  a. Anti-EBNA IgG titer (top quartile is 640 and 2560 titers vs rest).  b. 25(OH)D is seasonally adjusted, per 10nmol/L, reversed for direction of MS risk.  c. Low sun exposure in Ausimmune is defined from self-reported sun exposure less than 2 hours/day in summer or winter in 3 years preceding FCD.  *d.* *HLA-DRB1*1501* risk genotype (rs3135388) is AA/AG vs GG (risk) .  e. EBNA IgG peptide, dichotomized at median (6716).  f. Low sun exposure in EIMS is defined from responses to three self-reported questions regarding sun exposure (frequency of sunbathing, frequency of recent travel to a sunnier country than Sweden, and frequency of use of sunbeds), estimating a composite score ranging 3-12, here dichotomized and low sun exposure defined as sun score≤6.  Results in boldface denotes statistical significance (p<0.05). | | | | | | |

**Supplemental Table S6**. Sensitivity Analysis - Ausimmune: Mediation of selected environmental/lifestyle factors by A1-module and A2-module, restricted to FDEs.

|  | Total effect | | Direct effect | | | Indirect effect | | Percent indirect effect |
| --- | --- | --- | --- | --- | --- | --- | --- | --- |
|  | aOR (95%CI) | p-value | aOR (95%CI) | p-value | aOR (95%CI) | | p-value |  |
| A1-module |  |  |  |  |  | |  |  |
| History of Infectious Mononucleosis (yes vs no) | 2.41 (1.41, 4.12) | 0.001 | 1.83 (1.13, 2.96) | 0.014 | 1.32 (1.03, 1.69) | | 0.031 | 32.0% |
| Anti-EBNA IgG titer (top quartile vs rest)^a^ | 2.18 (1.17, 4.04) | 0.014 | 1.50 (0.87, 2.62) | 0.15 | 1.45 (1.07, 1.96) | | 0.017 | 47.0% |
| Lower 25(OH)D^b^ | 1.05 (0.97, 1.14) | 0.26 | 1.02 (0.96, 1.1) | 0.48 | 1.02 (0.98, 1.06) | | 0.27 | 50.0% |
| Low summer sun^c^ | 1.52 (0.95, 2.43) | 0.081 | 1.20 (0.80, 1.82) | 0.38 | 1.26 (1.03, 1.55) | | 0.027 | 56.0% |
| *HLA-DRB1*1501* (rs3135388, AA/AG vs GG)^d^ | 2.96 (1.84, 4.74) | <0.001 | 2.34 (1.53, 3.58) | <0.001 | 1.26 (1.03, 1.56) | | 0.026 | 21.0% |
| A2-module |  |  |  |  |  | |  |  |
| History of Infectious Mononucleosis (yes vs no) | 2.43 (1.41, 4.16) | 0.001 | 2.14 (1.29, 3.54) | 0.003 | 1.14 (0.92, 1.41) | | 0.24 | 15.0% |
| Anti-EBNA IgG titer (top quartile vs rest)^e^ | 2.16 (1.18, 3.98) | 0.013 | 1.65 (0.92, 2.94) | 0.09 | 1.31 (1.02, 1.68) | | 0.033 | 35.0% |
| Lower 25(OH)D^b^ | 1.05 (0.97, 1.13) | 0.26 | 1.03 (0.95, 1.11) | 0.49 | 1.02 (0.99, 1.05) | | 0.19 | 40.0% |
| Low summer sun^c^ | 1.51 (0.95, 2.4) | 0.079 | 1.25 (0.81, 1.92) | 0.32 | 1.21 (1.02, 1.45) | | 0.032 | 46.0% |
| *HLA-DRB1*1501* (rs3135388, AA/AG vs GG)^d^ | 2.97 (1.85, 4.76) | <0.001 | 2.43 (1.56, 3.77) | <0.001 | 1.23 (1.01, 1.48) | | 0.036 | 18.0% |
| Abbreviations: aOR=adjusted odds ratio; 95%CI=95% confidence interval; 25(OH)D=25-hydroxyvitamin D; EBNA=Epstein Barr Nuclear Antigen; FDE=first demyelinating event; HLA=Human Leucocyte Antigen.  a. Anti-EBNA IgG titer (top quartile is 640 and 2560 titers vs rest).  b. 25(OH)D is seasonally adjusted, per 10nmol/L, reversed for direction of MS risk.  c. Low sun exposure in Ausimmune is defined from self-reported sun exposure less than 2 hours/day in summer or winter in 3 years preceding FCD.  *d. HLA-DRB1*1501* risk genotype (rs3135388) is AA/AG vs GG (risk).  All analyses undertaken using logistic regression, with indirect effects estimated using the *medflex* package in R. All analyses adjusted for age, sex, and study region. Results in boldface denote statistical significance (p<0.05). | | | | | | | | |

**Supplemental Table S7**. Sensitivity analysis - Ausimmune: Mediation of selected environmental/lifestyle factors by A1-module and A2-module DNAm modules, restricted to cases not being treated with disease-modifying therapies (DMTs) at time of survey.

|  | Total effect | | Direct effect | | Indirect effect | | Percent indirect effect |
| --- | --- | --- | --- | --- | --- | --- | --- |
|  | aOR (95%CI) | p-value | aOR (95%CI) | p-value | aOR (95%CI) | p-value |  |
| A1-module |  |  |  |  |  |  |  |
| History of Infectious Mononucleosis (yes vs no) | 2.21 (1.39, 3.53) | <0.001 | 1.69 (1.12, 2.54) | 0.012 | 1.31 (1.04, 1.65) | 0.020 | 34.0% |
| Anti-EBNA IgG titer (top quartile vs rest)^a^ | 2.08 (1.21, 3.57) | 0.008 | 1.59 (0.98, 2.57) | 0.062 | 1.31 (1.01, 1.70) | 0.042 | 37.0% |
| Lower 25(OH)D^b^ | 1.57 (0.84, 2.93) | 0.16 | 1.20 (0.70, 2.06) | 0.50 | 1.31 (0.96, 1.77) | 0.084 | 60.0% |
| Low summer sun^c^ | 1.63 (1.10, 2.42) | 0.015 | 1.30 (0.92, 1.83 | 0.13 | 1.25 (1.03, 1.52) | 0.021 | 47.0% |
| *HLA-DRB1*1501* (rs3135388, AA/AG vs GG)^d^ | 2.91 (1.96, 4.31) | 0.001 | 2.24 (1.58, 3.17) | <0.001 | 1.30 (1.07, 1.57) | 0.007 | 24.0% |
| A2-module |  |  |  |  |  |  |  |
| History of Infectious Mononucleosis (yes vs no) | 2.26 (1.40, 3.64) | <0.001 | 1.97 (1.28, 3.05) | 0.002 | 1.14 (0.92, 1.43) | 0.23 | 16.0% |
| Anti-EBNA IgG titer (top quartile vs rest)^a^ | 2.06 (1.21, 3.49) | 0.008 | 1.67 (1.04, 2.71) | 0.035 | 1.23 (0.98, 1.54) | 0.074 | 28.0% |
| Lower 25(OH)D^b^ | 1.55 (0.86, 2.77) | 0.14 | 1.46 (0.,87, 2.46) | 0.15 | 1.06 (0.83, 1.35) | 0.65 | 14.0% |
| Low summer sun^c^ | 1.62 (1.10, 2.39) | 0.014 | 1.30 (0.91, 1.85) | 0.15 | 1.25 (1.05, 1.49) | 0.012 | 46.0% |
| *HLA-DRB1*1501* (rs3135388, AA/AG vs GG)^d^ | 2.92 (1.97, 4.33) | <0.001 | 2.37 (1.67, 3.38) | <0.001 | 1.23 (1.03, 1.46) | 0.019 | 20.0% |
| Abbreviations: aOR=adjusted odds ratio; 95%CI=95% confidence interval; 25(OH)D=25-hydroxyvitamin D; EBNA=Epstein Barr Nuclear Antigen; HLA=Human Leucocyte Antigen.  a. Anti-EBNA IgG titer (top quartile is 640 and 2560 titers vs rest).  b. 25(OH)D is seasonally adjusted, per 10nmol/L, reversed for direction of MS risk.  c. Low sun exposure in Ausimmune is defined from self-reported sun exposure less than 2 hours/day in summer or winter in 3 years preceding FCD.  *d. HLA-DRB1*1501* risk genotype (rs3135388) is AA/AG vs GG (risk).  All analyses undertaken using logistic regression, with indirect effects estimated using the *medflex* package in R. All analyses adjusted for age, sex, and study region. Results in boldface denote statistical significance (p<0.05). | | | | | | | |

**Supplemental Table S8.** EIMS: Mediation of exposure-MS associations by DNAm modules.

|  | Total effect | | Direct effect | | Indirect effect | | Percent indirect effect |
| --- | --- | --- | --- | --- | --- | --- | --- |
|  | aOR (95%CI) | p-value | aOR (95%CI) | p-value | aOR (95%CI) | p-value |  |
| E1-module |  |  |  |  |  |  |  |
| History of Infectious Mononucleosis (yes vs no) | 1.59 (0.94, 2.69) | 0.083 | 1.39 (0.90, 2.15) | 0.13 | 1.14 (0.92, 1.42) | 0.23 | 28.0% |
| Anti-EBNA IgG peptide (above median vs rest)^a^ | 3.08 (1.83, 5.18) | <0.001 | 1.99 (1.25, 3.17) | 0.004 | 1.55 (1.22, 1.96) | <0.001 | 39.0% |
| Lower 25(OH)D^b^ | 1.17 (1.06, 1.3) | 0.003 | 1.12 (1.02, 1.23) | 0.016 | 1.04 (1.00, 1.09) | 0.077 | 25.0% |
| Low sun-seeking behaviors^c^ | 1.03 (0.63, 1.69) | 0.91 | 0.87 (0.55, 1.37) | 0.55 | 1.18 (0.94, 1.49) | 0.15 | - |
| *HLA-DRB1*1501* (rs3135388, AA/AG vs GG)^d^ | 3.79 (2.22, 6.48) | <0.001 | 1.97 (1.17, 3.3) | 0.011 | 1.93 (1.48, 2.51) | <0.001 | 49.0% |
| E2-module |  |  |  |  |  |  |  |
| History of Infectious Mononucleosis (yes vs no) | 1.52 (0.96, 2.39) | 0.073 | 1.49 (0.96, 2.29) | 0.074 | 1.02 (0.86, 1.2) | 0.82 | 5.0% |
| Anti-EBNA IgG peptide (above median vs rest)^a^ | 3.09 (1.83, 5.21) | <0.001 | 2.62 (1.59, 4.32) | <0.001 | 1.18 (0.99, 1.41) | 0.072 | 15.0% |
| Lower 25(OH)D^b^ | 1.17 (1.05, 1.30) | 0.003 | 1.13 (1.02, 1.25) | 0.015 | 1.03 (1.00, 1.07) | 0.079 | 20.0% |
| Low sun-seeking behaviors^c^ | 1.03 (0.62, 1.69) | 0.91 | 1.19 (0.74, 1.9) | 0.48 | 0.87 (0.72, 1.04) | 0.12 | - |
| *HLA-DRB1*1501* (rs3135388, AA/AG vs GG)^d^ | 3.78 (2.22, 6.45) | <0.001 | 1.88 (0.96, 3.69) | 0.067 | 2.01 (1.27, 3.19) | 0.003 | 53.0% |
| Abbreviations: aOR=adjusted odds ratio; 95%CI=95% confidence interval; 25(OH)D=25-hydroxyvitamin D; EBNA=Epstein Barr Nuclear Antigen; HLA=Human Leucocyte Antigen.  a. Anti-EBNA IgG titer dichotomized as above median vs rest.  b. 25(OH)D is seasonally adjusted, per 10nmol/L, reversed for direction of MS risk.  c. Low sun exposure in EIMS is defined from responses to three self-reported questions regarding sun exposure (frequency of sunbathing, frequency of recent travel to a sunnier country than Sweden, and frequency of use of sunbeds), estimating a composite score ranging 3-12, here dichotomized and low sun exposure defined as sun score≤6.  *d. HLA-DRB1*1501* risk genotype (rs3135388) is AA/AG vs GG (risk).  All analyses undertaken using logistic regression, with indirect effects estimated using the *medflex* package in R. All analyses adjusted for age, sex, ethnicity, and smoking status. Results in boldface denote statistical significance (p<0.05). Mediation was also observed when using a different approach, that of the historical Küpers et al 2015^1^. | | | | | | | |

**Supplemental Table S9.** Sensitivity analysis - EIMS: Mediation of exposure-MS associations by DNAm modules, restricted to cases not being treated with disease-modifying therapies (DMTs) at time of survey.

|  | Total effect | | Direct effect | | Indirect effect | | | Percent indirect effect |
| --- | --- | --- | --- | --- | --- | --- | --- | --- |
|  | aOR (95%CI) | p-value | aOR (95%CI) | p-value | aOR (95%CI) | p-value |  | |
| E1-module |  |  |  |  |  |  |  | |
| History of Infectious Mononucleosis (yes vs no) | 1.70 (0.74, 3.90) | 0.21 | 1.56 (0.78, 3.12) | 0.21 | 1.09 (0.79, 1.50) | 0.60 | 17.0% | |
| Anti-EBNA IgG peptide (above median vs rest)^a^ | 2.85 (1.35, 6.00) | 0.006 | 1.95 (0.98, 3.87) | 0.058 | 1.46 (1.08, 1.98) | 0.013 | 36.0% | |
| Lower 25(OH)D^b^ | 1.07 (0.91, 1.25) | 0.40 | 1.04 (0.90, 1.20) | 0.62 | 1.03 (0.97, 1.09) | 0.29 | 43.0% | |
| Low sun-seeking behaviors^c^ | 1.10 (0.54, 2.24) | 0.80 | 0.97 (0.50, 1.86) | 0.92 | 1.13 (0.85, 1.50) | 0.39 | - | |
| *HLA-DRB1*1501* (rs3135388, AA/AG vs GG)^d^ | 4.04 (1.98, 8.26) | <0.001 | 2.20 (1.07, 4.56) | 0.033 | 1.83 (1.27, 2.65) | 0.001 | 44.0% | |
| E2-module |  |  |  |  |  |  |  | |
| History of Infectious Mononucleosis (yes vs no) | 1.66 (0.75, 3.69) | 0.21 | 1.54 (0.72, 3.30) | 0.27 | 1.08 (0.83, 1.42) | 0.56 | 16.0% | |
| Anti-EBNA IgG peptide (above median vs rest)^a^ | 2.85 (1.37, 5.93) | 0.005 | 2.55 (1.24, 5.21) | 0.011 | 1.12 (0.90, 1.40) | 0.32 | 11.0% | |
| Lower 25(OH)D^b^ | 1.07 (0.92, 1.24) | 0.39 | 1.04 (0.90, 1.20) | 0.60 | 1.03 (0.98, 1.07) | 0.24 | 43.0% | |
| Low sun-seeking behaviors^c^ | 1.09 (0.53, 2.24) | 0.81 | 1.19 (0.60, 2.39) | 0.62 | 0.92 (0.74, 1.14) | 0.43 | - | |
| *HLA-DRB1*1501* (rs3135388, AA/AG vs GG)^d^ | 4.01 (1.98, 8.16) | <0.001 | 2.48 (1.00, 6.16) | 0.050 | 1.62 (0.90, 2.93) | 0.11 | 35.0% | |
| Abbreviations: aOR=adjusted odds ratio; 95%CI=95% confidence interval; 25(OH)D=25-hydroxyvitamin D; EBNA=Epstein Barr Nuclear Antigen; HLA=Human Leucocyte Antigen.  a. Anti-EBNA IgG titer dichotomized as above median vs rest.  b. 25(OH)D is seasonally adjusted, per 10nmol/L, reversed for direction of MS risk.  c. Low sun exposure in EIMS is defined from responses to three self-reported questions regarding sun exposure (frequency of sunbathing, frequency of recent travel to a sunnier country than Sweden, and frequency of use of sunbeds), estimating a composite score ranging 3-12, here dichotomized and low sun exposure defined as sun score≤6.  *d. HLA-DRB1*1501* risk genotype (rs3135388) is AA/AG vs GG (risk).  All analyses undertaken using logistic regression, with indirect effects estimated using the *medflex* package in R. All analyses adjusted for age, sex, ethnicity, and smoking status. Results in boldface denote statistical significance (p<0.05). | | | | | | | | |

**Supplemental Table S10.** Sensitivity analysis - EIMS: Mediation of exposure-MS associations by DNAm modules, restricted to cases up to 5 years post-MS onset at time of survey.

|  | Total effect | | Direct effect | | Indirect effect | | | Percent indirect effect |
| --- | --- | --- | --- | --- | --- | --- | --- | --- |
|  | aOR (95%CI) | p-value | aOR (95%CI) | p-value | aOR (95%CI) | p-value |  | |
| E1-module |  |  |  |  |  |  |  | |
| History of Infectious Mononucleosis (yes vs no) | 1.30 (0.37, 4.53) | 0.68 | 1.13 (0.37, 3.48) | 0.83 | 1.15 (0.81, 1.62) | 0.43 | 52.0% | |
| Anti-EBNA IgG peptide (above median vs rest)^+^ | 3.49 (1.72, 7.10) | <0.001 | 2.24 (1.15, 4.36) | 0.018 | 1.56 (1.17, 2.07) | 0.002 | 35.0% | |
| Lower 25(OH)D | 1.12 (0.96, 1.30) | 0.14 | 1.09 (0.95, 1.26) | 0.21 | 1.02 (0.97, 108) | 0.37 | 18.0% | |
| Low sun-seeking behaviours | 0.76 (0.39, 1.48) | 0.42 | 0.69 (0.37. 1.27) | 0.23 | 1.10 (0.86, 1.42) | 0.45 | - | |
| *HLA-DRB1*1501* (rs3135388, AA/AG vs GG) | 5.09 (2.47, 10.48) | <0.001 | 2.95 (1.42, 6.13) | 0.004 | 1.72 (1.24, 2.40) | 0.001 | 33.0% | |
| E2-module |  |  |  |  |  |  |  | |
| History of Infectious Mononucleosis (yes vs no) | 1.29 (0.35, 4.78) | 0.70 | 1.09 (0.31, 3.75) | 0.90 | 1.19 (0.89, 1.59) | 0.24 | 68.0% | |
| Anti-EBNA IgG peptide (above median vs rest) ^+^ | 3.48 (1.72, 7.03) | <0.001 | 2.82 (1.48, 5.37) | 0.002 | 1.23 (0.94, 1.61) | 0.12 | 17.0% | |
| Lower 25(OH)D | 1.12 (0.96, 1.29) | 0.14 | 1.09 (0.95, 1.25) | 0.21 | 1.02 (0.97, 1.08) | 0.44 | 18.0% | |
| Low sun-seeking behaviours | 0.76 (0.39, 1.48) | 0.42 | 0.88 (0.48, 1.61) | 0.67 | 0.86 (0.67, 1.12) | 0.27 | 54.0% | |
| *HLA-DRB1*1501* (rs3135388, AA/AG vs GG) | 5.07 (2.47, 10.42) | <0.001 | 2.16 (0.89, 5.25) | 0.089 | 2.35 (1.23, 4.47) | 0.009 | 52.0% | |
| Abbreviations: aOR=adjusted odds ratio; 95%CI=95% confidence interval; 25(OH)D=25-hydroxyvitamin D; EBNA=Epstein Barr Nuclear Antigen; HLA=Human Leucocyte Antigen.  a. Anti-EBNA IgG titer dichotomized as above median vs rest.  b. 25(OH)D is seasonally adjusted, per 10nmol/L, reversed for direction of MS risk.  c. Low sun exposure in EIMS is defined from responses to three self-reported questions regarding sun exposure (frequency of sunbathing, frequency of recent travel to a sunnier country than Sweden, and frequency of use of sunbeds), estimating a composite score ranging 3-12, here dichotomized and low sun exposure defined as sun score≤6.  *d. HLA-DRB1*1501* risk genotype (rs3135388) is AA/AG vs GG (risk).  All analyses undertaken using logistic regression, with indirect effects estimated using the *medflex* package in R. All analyses adjusted for age, sex, and smoking status. Results in boldface denote statistical significance (p<0.05). | | | | | | | | |

**Supplemental Table S11**. Sensitivity analysis, Ausimmune- reverse (MS-to-risk factor) mediation

|  | Total effect | | Direct effect | | Indirect effect | | Percent indirect effect |
| --- | --- | --- | --- | --- | --- | --- | --- |
|  | aOR (95%CI) | p-value | aOR (95%CI) | p-value | aOR (95%CI) | p-value |  |
| History of Infectious Mononucleosis | | | | | | | |
| A1-module | 2.4 (1.55, 3.71) | 8.10 × 10^-5^ | 2.17 (1.33, 3.55) | 0.002 | 1.11 (0.85, 1.44) | 0.460 | 11.0% |
| A2-module | 2.4 (1.55, 3.71) | 8.10 × 10^-5^ | 2.53 (1.56, 4.11) | 1.6 × 10^-4^ | 0.95 (0.76, 1.18) | 0.630 | - |
| Anti-EBNA IgG titer (top quartile is 640 and 2560 titers vs rest)^a^ | | | | | | | |
| A1-module | 2.02 (1.18, 3.45) | 0.010 | 1.75 (0.95, 3.25) | 0.074 | 1.15 (0.85, 1.57) | 0.370 | 20.0% |
| A2-module | 2.02 (1.18, 3.45) | 0.010 | 1.94 (1.07, 3.52) | 0.030 | 1.04 (0.82, 1.32) | 0.740 | 6.0% |
| Low 25(OH)D^b^ | | | | | | | |
| A1-module | 1.89 (1.17, 3.04) | 0.009 | 1.61 (0.95, 2.73) | 0.076 | 1.17 (0.9, 1.52) | 0.240 | 25.0% |
| A2-module | 1.89 (1.17, 3.04) | 0.009 | 1.65 (0.95, 2.85) | 0.075 | 1.15 (0.92, 1.43) | 0.230 | 22.0% |
| Low summer sun^c^ | | | | | | | |
| A1-module | 1.73 (1.22, 2.46) | 0.002 | 1.46 (0.97, 2.19) | 0.069 | 1.19 (0.97, 1.46) | 0.100 | 31.0% |
| A2-module | 1.73 (1.22, 2.46) | 0.002 | 1.45 (0.97, 2.16) | 0.068 | 1.19 (1, 1.43) | 0.052 | 33.0% |
| Abbreviations: 25(OH)D=25-hydroxyvitamin D; EBNA=Epstein Barr Nuclear Antigen; HLA=Human Leukocyte Antigen.  a. Anti-EBNA IgG titer (top quartile is 640 and 2560 titers vs rest).  b. 25(OH)D is seasonally adjusted, per 10nmol/L, reversed for direction of MS risk.  c. Low sun exposure in Ausimmune is defined from self-reported sun exposure less than 2 hours/day in summer or winter in 3 years preceding FCD.  Indirect effects estimated using the *medflex* package in R. All analyses adjusted for age, sex, and region. Results in boldface denote statistical significance (p<0.05). | | | | | | | |

**Supplemental Table S12.** Sensitivity analysis, EIMS-reverse (MS-to-risk factor) mediation

|  | Total effect | | Direct effect | | Indirect effect | | Percent indirect effect |
| --- | --- | --- | --- | --- | --- | --- | --- |
|  | OR (95%CI) | p-value | OR (95%CI) | p-value | OR (95%CI) | p-value |  |
| History of Infectious Mononucleosis (yes vs no) | | | | | | | |
| E1-module | 1.12 (1.00, 1.27) | 0.060 | 1.11 (0.99, 1.25) | 0.071 | 1.01 (0.96, 1.07) | 0.720 | 8.0% |
| E2-module | 1.12 (1.00, 1.27) | 0.060 | 1.13 (0.98, 1.31) | 0.083 | 0.99 (0.94, 1.04) | 0.720 | - |
| Anti-EBNA IgG peptide (above median vs rest)^a^ | | | | | | | |
| E1-module | 3.08 (1.83, 5.19) | 2.20 × 10^-5^ | 2.34 (1.33, 4.13) | 0.003 | 1.32 (1, 1.73) | 0.051 | 24.0% |
| E2-module | 3.08 (1.83, 5.18) | 2.20 × 10^-5^ | 2.97 (1.7, 5.17) | 1.2 × 10^-4^ | 1.04 (0.86, 1.26) | 0.700 | 4.0% |
| Lower 25(OH)D^b^ | | | | | | | |
| E1-module | 2.54 (1.42, 4.54) | 0.002 | 2.41 (1.24, 4.71) | 0.001 | 1.05 (0.77, 1.44) | 0.760 | 5.0% |
| E2-module | 2.54 (1.42, 4.54) | 0.002 | 2.25 (1.21, 4.18) | 0.011 | 1.13 (0.9, 1.42) | 0.300 | 13.0% |
| Low sun-seeking behaviors^c^ | | | | | | | |
| E1-module | 1.03 (0.62, 1.69) | 0.910 | 0.84 (0.48, 1.48) | 0.540 | 1.23 (0.95, 1.59) | 0.120 | - |
| E2-module | 1.03 (0.62, 1.69) | 0.910 | 1.2 (0.71, 2.03) | 0.490 | 0.86 (0.71, 1.03) | 0.110 | - |
| Abbreviations: aOR=adjusted odds ratio; 95%CI=95% confidence interval; 25(OH)D=25-hydroxyvitamin D; EBNA=Epstein Barr Nuclear Antigen; HLA=Human Leucocyte Antigen.  a. Anti-EBNA IgG titer dichotomized as above median vs rest.  b. 25(OH)D is seasonally adjusted, per 10nmol/L, reversed for direction of MS risk.  c. Low sun exposure in EIMS is defined from responses to three self-reported questions regarding sun exposure (frequency of sunbathing, frequency of recent travel to a sunnier country than Sweden, and frequency of use of sunbeds), estimating a composite score ranging 3-12, here dichotomized and low sun exposure defined as sun score≤6.  Indirect effects estimated using the *medflex* package in R. All analyses adjusted for age, sex, and smoking status. Results in boldface denote statistical significance (p<0.05). | | | | | | | |

Supplemental Table S13. pathfindR enrichment results for pathways common to Ausimmune and EIMS DNAm modules: Reactome.

| ID | Term | Fold Enrichment | Support | Highest P-value | Term rank in set | Status | Grouping |
| --- | --- | --- | --- | --- | --- | --- | --- |
| R-HSA-69620 | Cell Cycle Checkpoints |  |  |  |  |  |  |
|  | A1-module | - | - | - | - | - | - |
|  | A2-module | 1.04 | 0.0103 | 0.0278 | 179 | Representative | 198 |
|  | E1-module | 1.2 | 0.00568 | 0.00292 | 81 | Representative | 52 |
|  | E2-module | 1.92 | 0.035 | 0.000374 | 64 | Member | 8 |
| R-HSA-3247509 | Chromatin modifying enzymes |  |  |  |  |  |  |
|  | A1-module | 1.78 | 0.0132 | 0.0306 | 119 | Representative | 24 |
|  | A2-module | 1.88 | 0.0387 | 0.00165 | 19 | Representative | 25 |
|  | E1-module | 1.32 | 0.0155 | 0.000235 | 39 | Representative | 34 |
|  | E2-module | 1.93 | 0.00767 | 0.0431 | 104 | Representative | 69 |
| R-HSA-4839726 | Chromatin organization |  |  |  |  |  |  |
|  | A1-module | 1.78 | 0.0132 | 0.0306 | 120 | Member | 24 |
|  | A2-module | 1.88 | 0.0387 | 0.00165 | 20 | Member | 25 |
|  | E1-module | 1.32 | 0.0155 | 0.000235 | 40 | Member | 34 |
|  | E2-module | 1.93 | 0.00767 | 0.0431 | 105 | Member | 69 |
| R-HSA-5621481 | C-type lectin receptors (CLRs) |  |  |  |  |  |  |
|  | A1-module | 1.28 | 0.0181 | 0.00527 | 51 | Member | 58 |
|  | A2-module | 1.89 | 0.0218 | 0.00274 | 68 | Representative | 76 |
|  | E1-module | 1.09 | 0.0346 | 1.86E-05 | 22 | Representative | 17 |
|  | E2-module | 2.77 | 0.04 | 8.18E-05 | 40 | Member | 13 |
| R-HSA-983231 | Factors involved in megakaryocyte development and platelet production |  |  |  |  |  |  |
|  | A1-module | - | - | - | - | - | - |
|  | A2-module | 2.81 | 0.0109 | 0.0261 | 167 | Representative | 206 |
|  | E1-module | 2.84 | 0.00556 | 0.049 | 176 | Representative | 83 |
|  | E2-module | 2.17 | 0.01 | 0.0298 | 99 | Representative | 83 |
| R-HSA-2454202 | Fc epsilon receptor (FCERI) signalling |  |  |  |  |  |  |
|  | A1-module | 1.4 | 0.0058 | 0.0472 | 151 | Member | 121 |
|  | A2-module | 1.1 | 0.0105 | 0.0415 | 220 | Member | 129 |
|  | E1-module | 0.667 | 0.0293 | 0.00846 | 109 | Member | 17 |
|  | E2-module | 2.27 | 0.04 | 0.00133 | 49 | Member | 13 |
| R-HSA-909733 | Interferon alpha/beta signalling |  |  |  |  |  |  |
|  | A1-module | - | - | - | - | - | - |
|  | A2-module | 1.55 | 0.00524 | 0.0288 | 181 | Representative | 213 |
|  | E1-module | 3.65 | 0.0109 | 0.000354 | 50 | Representative | 13 |
|  | E2-module | 10.6 | 0.0325 | 0.000532 | 68 | Representative | 34 |
| R-HSA-913531 | Interferon signaling |  |  |  |  |  |  |
|  | A1-module | - | - | - | - | - | - |
|  | A2-module | 1.11 | 0.00518 | 0.00954 | 116 | Representative | 145 |
|  | E1-module | 2.81 | 0.0103 | 0.000354 | 51 | Representative | 15 |
|  | E2-module | 7.17 | 0.0475 | 9.30E-10 | 6 | Member | 2 |
| R-HSA-5684996 | MAPK1/MAPK3 signaling |  |  |  |  |  |  |
|  | A1-module | - | - | - | - | - | - |
|  | A2-module | 1.48 | 0.0421 | 0.000701 | 39 | Member | 9 |
|  | E1-module | 1.62 | 0.0178 | 0.0061 | 98 | Representative | 65 |
|  | E2-module | 2.76 | 0.035 | 0.000503 | 66 | Member | 26 |
| R-HSA-453274 | Mitotic G2-G2/M phases |  |  |  |  |  |  |
|  | A1-module | - | - | - | - | - | - |
|  | A2-module | 0.618 | 0.00529 | 0.00278 | 71 | Member | 28 |
|  | E1-module | 0.831 | 0.00568 | 0.0146 | 128 | Member | 108 |
|  | E2-module | 1.59 | 0.03 | 0.00155 | 54 | Member | 5 |
| R-HSA-69275 | G2/M Transition |  |  |  |  |  |  |
|  | A1-module | 1.32 | 0.0116 | 0.011 | 80 | Member | 43 |
|  | A2-module | 0.625 | 0.00529 | 0.00278 | 70 | Member | 28 |
|  | E1-module | 0.841 | 0.00568 | 0.0146 | 127 | Representative | 108 |
|  | E2-module | 1.61 | 0.03 | 0.00153 | 53 | Member | 5 |
| R-HSA-76002 | Platelet activation, signaling and aggregation |  |  |  |  |  |  |
|  | A1-module | 2.12 | 0.0213 | 0.0202 | 108 | Representative | 48 |
|  | A2-module | 1.48 | 0.0472 | 0.00289 | 72 | Representative | 51 |
|  | E1-module | 2.25 | 0.0763 | 1.07E-07 | 3 | Representative | 3 |
|  | E2-module | 2.29 | 0.0125 | 0.0335 | 100 | Representative | 49 |
| R-HSA-9013149 | RAC1 GTPase cycle |  |  |  |  |  |  |
|  | A1-module | 3.96 | 0.0236 | 0.0306 | 118 | Representative | 61 |
|  | A2-module | 1.88 | 0.00539 | 0.00384 | 76 | Representative | 110 |
|  | E1-module | 1.89 | 0.0132 | 0.01 | 110 | Representative | 1 |
|  | E2-module | 3.22 | 0.01 | 0.0335 | 101 | Representative | 31 |
| R-HSA-5673001 | RAF/MAP kinase cascade |  |  |  |  |  |  |
|  | A1-module | - | - | - | - | - | - |
|  | A2-module | 1.37 | 0.0368 | 0.000701 | 38 | Representative | 9 |
|  | E1-module | 1.52 | 0.0127 | 0.0496 | 178 | Member | 65 |
|  | E2-module | 2.82 | 0.035 | 0.000476 | 65 | Representative | 26 |
| R-HSA-9013408 | RHOG GTPase cycle |  |  |  |  |  |  |
|  | A1-module | - | - | - | - | - | - |
|  | A2-module | 1.57 | 0.0108 | 0.0116 | 122 | Representative | 156 |
|  | E1-module | 2.64 | 0.00517 | 0.0074 | 106 | Representative | 21 |
|  | E2-module | 4.04 | 0.005 | 0.0135 | 91 | Representative | 71 |
| R-HSA-8939236 | RUNX1 regulates transcription of genes involved in differentiation of HSCs |  |  |  |  |  |  |
|  | A1-module | - | - | - | - | - | - |
|  | A2-module | 1.62 | 0.00544 | 0.0271 | 172 | Representative | 191 |
|  | E1-module | 2.17 | 0.0107 | 0.0159 | 130 | Representative | 88 |
|  | E2-module | 4.16 | 0.03 | 2.06E-05 | 17 | Representative | 6 |
| R-HSA-2467813 | Separation of Sister Chromatids |  |  |  |  |  |  |
|  | A1-module | - | - | - | - | - | - |
|  | A2-module | 1.36 | 0.0104 | 0.014 | 131 | Member | 97 |
|  | E1-module | 0.683 | 0.00518 | 0.0308 | 157 | Member | 101 |
|  | E2-module | 1.74 | 0.03 | 0.00129 | 46 | Member | 9 |
| R-HSA-157118 | Signaling by NOTCH |  |  |  |  |  |  |
|  | A1-module | 3.26 | 0.0522 | 0.00271 | 37 | Representative | 22 |
|  | A2-module | 2.06 | 0.0104 | 0.0296 | 183 | Representative | 159 |
|  | E1-module | 1.87 | 0.0284 | 0.00622 | 99 | Member | 35 |
|  | E2-module | 3.18 | 0.03 | 0.00155 | 55 | Representative | 30 |
| R-HSA-983705 | signaling by the B Cell Receptor (BCR) |  |  |  |  |  |  |
|  | A1-module | 1.58 | 0.0058 | 0.0367 | 134 | Representative | 121 |
|  | A2-module | 1.25 | 0.00518 | 0.0153 | 144 | Member | 129 |
|  | E1-module | 1.51 | 0.0411 | 0.000543 | 53 | Member | 4 |
|  | E2-module | 2.57 | 0.04 | 9.8E3-05 | 42 | Member | 13 |
| R-HSA-201681 | TCF dependent signaling in response to WNT |  |  |  |  |  |  |
|  | A1-module | 1.04 | 0.0118 | 0.0335 | 128 | Representative | 31 |
|  | A2-module | 1.09 | 0.0051 | 0.0353 | 204 | Representative | 229 |
|  | E1-module | 1.32 | 0.0107 | 0.00126 | 63 | Member | 9 |
|  | E2-module | 2.24 | 0.03 | 0.00135 | 50 | Member | 12 |
| R-HSA-8878171 | Transcriptional regulation by RUNX1 |  |  |  |  |  |  |
|  | A1-module | 1.62 | 0.016 | 0.00753 | 66 | Representative | 75 |
|  | A2-module | 1.84 | 0.0207 | 0.00165 | 59 | Representative | 65 |
|  | E1-module | 1.65 | 0.0214 | 0.00154 | 65 | Representative | 51 |
|  | E2-module | 2.11 | 0.03 | 0.00156 | 56 | Member | 6 |
| Term rank: the ranking of the term in each respective cluster set, ordered by p-value. The lower the number, the higher the significance rankings this pathway is in the cluster. Grouping: the number of the Grouping the pathway belongs to in the relationships of pathways for the epigenetic cluster. Status: Representative - the term is the representative pathway in a Grouping; Member - the term is a member of the Grouping. Again, lower number=higher ranked in terms of significance. Overall, 21 pathways were common across both studies’ epigenetic gene-sets. | | | | | | | |

**Supplemental Table S14**. pathfindR enrichment results for pathways common to Ausimmune and EIMS DNAm modules: Gene Ontology.

| ID | Term | Fold Enrichment | Support | Highest P-value | Term rank in set | Status | Grouping |
| --- | --- | --- | --- | --- | --- | --- | --- |
| GO:0004674 | Protein serine/threonine kinase activity |  |  |  |  |  |  |
|  | A1-module | 2.93 | 0.0227 | 1.97E-06 | 2 | Representative | 2 |
|  | A2-module | 2.31 | 0.0298 | 5.75E-06 | 2 | Representative | 2 |
|  | E1-module | 1.55 | 0.00786 | 0.00147 | 58 | Member | 13 |
|  | E2-module | 3.17 | 0.01 | 0.018 | 38 | Representative | 16 |
| GO:0046777 | Protein autophosphorylation |  |  |  |  |  |  |
|  | A1-module | 4.09 | 0.0262 | 1.97E-06 | 1 | Representative | 1 |
|  | A2-module | 3.1 | 0.026 | 3.28E-05 | 5 | Member | 2 |
|  | E1-module | 1.82 | 0.0252 | 0.00142 | 57 | Member | 14 |
|  | E2-module | 3.32 | 0.01 | 0.0334 | 46 | Member | 16 |
| GO:0051015 | Actin filament binding |  |  |  |  |  |  |
|  | A1-module | 4.45 | 0.00602 | 0.0123 | 24 | Member | 10 |
|  | A2-module | 2.81 | 0.0154 | 0.00219 | 19 | Representative | 7 |
|  | E1-module | 2.43 | 0.0214 | 0.00295 | 76 | Member | 4 |
|  | E2-module | 5.16 | 0.01 | 0.00845 | 25 | Representative | 12 |
| GO:0005925 | Focal adhesion |  |  |  |  |  |  |
|  | A1-module | - | - | - | - | - | - |
|  | A2-module | 1.97 | 0.00568 | 0.0145 | 52 | Representative | 5 |
|  | E1-module | 1.99 | 0.00517 | 0.00284 | 74 | Representative | 44 |
|  | E2-module | 3.62 | 0.005 | 4.68E-05 | 12 | Representative | 5 |
| GO:0005096 | GTPase activator activity |  |  |  |  |  |  |
|  | A1-module | - | - | - | - | - | - |
|  | A2-module | 2.51 | 0.00518 | 0.0342 | 80 | Member | 58 |
|  | E1-module | 2.82 | 0.00517 | 0.0428 | 173 | Member | 30 |
|  | E2-module | 5.75 | 0.005 | 0.0476 | 56 | Representative | 43 |
| Term rank: the ranking of the term in each respective cluster set, ordered by p-value. The lower the number, the higher the significance rankings this pathway is in the cluster. Grouping: the number of the Grouping the pathway belongs to in the relationships of pathways for the epigenetic cluster. Status: Representative - the term is the representative pathway in a Grouping; Member - the term is a member of the Grouping. Again, lower number=higher ranked in terms of significance. Overall, five pathways were common across both studies’ epigenetic gene-sets. | | | | | | | |

Supplemental references

1. Küpers LK, Xu X, Jankipersadsing SA, et al. DNA methylation mediates the effect of maternal smoking during pregnancy on birthweight of the offspring. *Int J Epidemiol.* 2015;44(4):1224-1237.
