# Supplementary material for: Epstein–Barr Virus, Lower Vitamin D, Low Sun Exposure, and HLA‐DRB1*1501 Risk Variant Share Common Epigenetic Pathways Leading to Multiple Sclerosis Onset
Source: Ann Neurol. 2025 Oct 10;99(2):341–55. doi: 10.1002/ana.78043 (PMC12894492; doi:10.1002/ana.78043)
Supplement: Supplementary file 2 — Supplementary Data S2 Ausimmune Investigators Group. [file ANA-99-341-s001.docx]

Ausimmune Investigators Group

| Investigator | Affiliation |
| --- | --- |
| Dr Caron Chapman | Barwon Health, Geelong, VIC, Australia |
| Professor Alan Coulthard | Royal Brisbane and Women's Hospital and the University of QLD, Brisbane, QLD, Australia |
| Professor Keith Dear | School of Public Health, University of Adelaide, Adelaide, SA, Australia |
| Professor Terry Dwyer | Murdoch Children’s Research Institute, The University of Melbourne, Melbourne, VIC, Australia |
| Professor Trevor Kilpatrick | Florey Institute of Neuroscience and Mental Health, The University of Melbourne, Melbourne, VIC, Australia |
| Professor Robyn Lucas | National Centre for Epidemiology and Population Health, Australian National University, Canberra, ACT, Australia |
| Professor Tony McMichael (deceased) | National Centre for Epidemiology and Population Health, Australian National University, Canberra, ACT, Australia |
| Professor Anne-Louise Ponsonby | Florey Institute of Neuroscience and Mental Health, The University of Melbourne, Melbourne, VIC, Australia |
| Professor Bruce Taylor | MS Research Flagship, Menzies Institute for Medical Research, University of Tasmania, Hobart, TAS, Australia |
| A/Professor Patricia Valery | QIMR Berghofer Medical Research Institute, Brisbane, QLD, Australia |
| Professor Ingrid van der Mei | Menzies Institute for Medical Research, University of Tasmania, Hobart, TAS, Australia |
| Dr David Williams | Hunter Health, Newcastle, NSW, Australia |
